# Investigation of Piezoresistive Behaviors in TPMS Lattice Structures Produced with MWCNT-Reinforced PLA Filament

**DOI:** 10.3390/polym18192449

**Published:** 2026-10-08

**Authors:** Mustafa Ercan Gündüz, Erkan Bahçe

**Affiliations:** 1 Department of Electrical and Energy, Malatya Organized Industrial Zone Vocational School, Inonu University, 44070 Malatya, Türkiye; 2 Department of Mechanical Engineering, Faculty of Engineering, Inonu University, 44280 Malatya, Türkiye

**Keywords:** additive manufacturing (AM), TPMS (triply periodic minimal surface), MWCNT/PLA composite, solvent mixing, piezoresistive sensors, gauge factor (GF)

## Abstract

Although the piezoresistive behavior of conductive polymer composites has been widely studied, the geometry-dependent piezoresistive response of MWCNT/PLA-based TPMS lattice structures produced by fused filament fabrication (FFF) remains insufficiently explored. The primary objective of this study is to comparatively investigate the mechanical, thermal, electrical, and piezoresistive behaviors of Gyroid (G), Diamond (D), and Schwarz Primitive (P) TPMS lattice structures produced using the same material system, constant relative density, and XY printing orientation. For this purpose, a 2 wt.% multi-walled carbon nanotube (MWCNT)-reinforced polylactic acid (PLA) composite filament was fabricated via solvent mixing and used to produce the three TPMS topologies through FFF. Mechanical and electromechanical properties were assessed via Shore D hardness, monotonic compression tests, and in-situ electrical measurements using three independent replicate specimens for each topology (*n* = 3). The highest hardness was recorded for Diamond (75.6 Shore D) and the lowest for Schwarz Primitive (71.2 Shore D). At 50% compressive strain, compressive stresses were 14.164, 8.777, and 3.152 MPa for Diamond, Gyroid, and Schwarz Primitive, respectively. Specific energy absorption (SEA) values followed the same order at 9.161, 6.149, and 2.917 J g^−1^. Electrical conductivity and resistivity variations under loading were characterized using the four-probe method, with Diamond exhibiting the highest and Schwarz Primitive the lowest conductivity. Piezoresistive analysis showed that Diamond achieved the highest positive compressive gauge factor (GF) values of 4.74 and 2.74 at 10% and 25% strains, respectively. At 50% strain, Gyroid demonstrated superior sensitivity (GF = 1.66) over Diamond (1.57) and Schwarz Primitive (1.49). Electrical resistance exhibited a monotonic decrease with advancing compressive strain across all topologies, validating a coherent and continuous piezoresistive response under uniaxial compression. These results indicate that Diamond offers the best overall performance in mechanical strength, SEA, and low-to-medium strain sensitivity, while Gyroid is preferable for high-strain sensing. Overall, 2 wt.% MWCNT/PLA-based TPMS structures demonstrate considerable potential for piezoresistive sensor applications.

## 1. Introduction

Additive manufacturing (AM) technologies have gained a significant place in engineering applications in recent years due to their ability to produce complex geometries with low material loss, enable personalized designs, and provide rapid prototyping [1,2,3,4]. Among these technologies, the fused filament fabrication (FFF) method has become one of the most widely used methods thanks to its advantages such as low cost, easy accessibility, and compatibility with thermoplastic-based materials [5]. Especially in the production of functional parts, providing not only mechanical performance but also electrical conductivity and sensor-based functionalities make FFF-based production even more important for the development of multifunctional structures [6]. Conductive polymer composites stand out as an important class of materials in the development of multifunctional structures [7]. Polylactic acid (PLA), one of the polymers preferred as a matrix material in the production of these composites, has a wide range of use due to its high processability and compatibility with FFF processes [8]. However, PLA in its pure form does not provide electrical conductivity. To overcome this problem, many additives such as metal-based reinforcements, fibers, and carbon-based nanoparticles are added to polymers [9,10]. In this field, carbon-based nanoparticles especially stand out with their performance and are the subject of many scientific studies today. Carbon-based nanoparticles are reinforced into polymers through methods such as melt blending, solution mixing, in-situ polymerization, sol–gel method, electrospinning, and coating/surface modification [11,12]. Among carbon-based nanoparticle types, multi-walled carbon nanotube (MWCNT) reinforcements stand out due to their extraordinary electrical and thermal properties [13,14]. MWCNTs increase the electrical conductivity and functional performance of the material when added to the PLA matrix, and the conductive network structure formed by MWCNT reinforcement undergoes changes under mechanical loading, causing measurable differences in the electrical resistance of the material; this situation forms the basis of piezoresistive behavior [15,16]. Piezoresistive behavior depends not only on the internal structure of the material but is also affected by parameters such as geometric design, load transfer mechanism, deformation mode, and cellular architecture. Therefore, even if the same material is used, significant differences can arise in the electro-mechanical responses of different lattice geometries [17]. At this point, Triply Periodic Minimal Surface (TPMS) based lattice structures stand out compared to traditional lattice topologies with their continuous curvilinear surfaces, low stress concentration, high specific strength, and energy dissipation potentials [18,19]. TPMS geometries contribute to a more homogeneous distribution of the load thanks to their structures showing continuity throughout the volume and exhibit more stable mechanical behavior during deformation [20]. Gyroid, Diamond, and Schwarz Primitive topologies are among the most studied lattice structures of the TPMS family and offer continuous curvilinear geometry, low stress concentration, and high surface area/volume ratio [21]. These structures differ from each other in terms of unit cell characteristics, surface continuity, void distribution, and deformation mechanisms, and these differences are decisive on piezoresistive behavior. Although there are numerous studies in the literature regarding the piezoresistive properties of conductive polymer composites, it is observed that studies comparatively examining the geometry-dependent piezoresistive behaviors of TPMS-based lattice structures produced by the FFF method using MWCNT-reinforced PLA filament are limited [22,23]. Particularly, evaluating Gyroid, Diamond, and Schwarz Primitive topologies under the same material system and similar production conditions is important in terms of revealing the relationship between geometry, mechanical performance, and electrical response. Such a comparison will contribute both to the determination of structural designs suitable for sensor applications and to the development of smart cellular materials. Recent studies have demonstrated the potential of material-extrusion-based CNT/polymer systems for multifunctional sensing applications. Syed et al. developed 3D-printed short carbon fiber/polypropylene sensors for real-time strain monitoring of large structures, demonstrating the relevance of extrusion-based conductive polymer systems for structural health-monitoring applications [24]. De Bortoli et al. investigated functionalized CNT-reinforced PLA nanocomposites produced by fused filament fabrication and evaluated the effects of CNT functionalization on the thermal and mechanical properties of the printed parts [25]. Similarly, Sam-Daliri et al. developed a CNT/Elium-based nanocomposite filament sensor and demonstrated its applicability for structural health monitoring through simultaneous mechanical and electrical measurements under cyclic loading [26]. These studies mainly focused on material composition, CNT functionalization, filament-based sensing, or integration into conventional composite structures. In contrast, the present study focuses on the effect of TPMS unit-cell topology on the mechanical, electrical, and piezoresistive responses of MWCNT/PLA lattice structures produced under identical material, relative-density, and processing conditions. Therefore, the comparison of Gyroid, Diamond, and Schwarz Primitive topologies provides a complementary perspective by linking the cellular architecture directly to the sensing performance of material-extrusion-produced conductive polymer structures.

The primary objective of this study is to systematically and comparatively investigate the geometry-dependent piezoresistive behavior of MWCNT/PLA-based TPMS lattice structures produced under identical material and processing conditions. In particular, the study examines the relationship between TPMS topology, mechanical deformation, electrical conductivity, and piezoresistive response. To this end, a low-percolation MWCNT-reinforced PLA filament was produced, and three different TPMS lattice structures Gyroid, Diamond, and Schwarz Primitive were fabricated in the XY orientation using the FFF method. The mechanical, thermal, electrical, and piezoresistive properties of the obtained structures were comprehensively investigated under a constant relative density. The novelty of this work lies in establishing a direct comparison between the topology-dependent mechanical performance and piezoresistive sensing response of Gyroid, Diamond, and Schwarz Primitive structures within the same MWCNT/PLA material system and under identical production conditions. The findings are expected to contribute to the development of suitable lattice designs for smart lightweight structures, pressure/strain sensors, and structural health-monitoring applications.

## 2. Materials and Methods

### 2.1. Materials

In this study, the solution mixing method was used to produce electrically conductive composite filament [27]. The materials used for filament production are summarized in Table 1.

**Figure 1:**
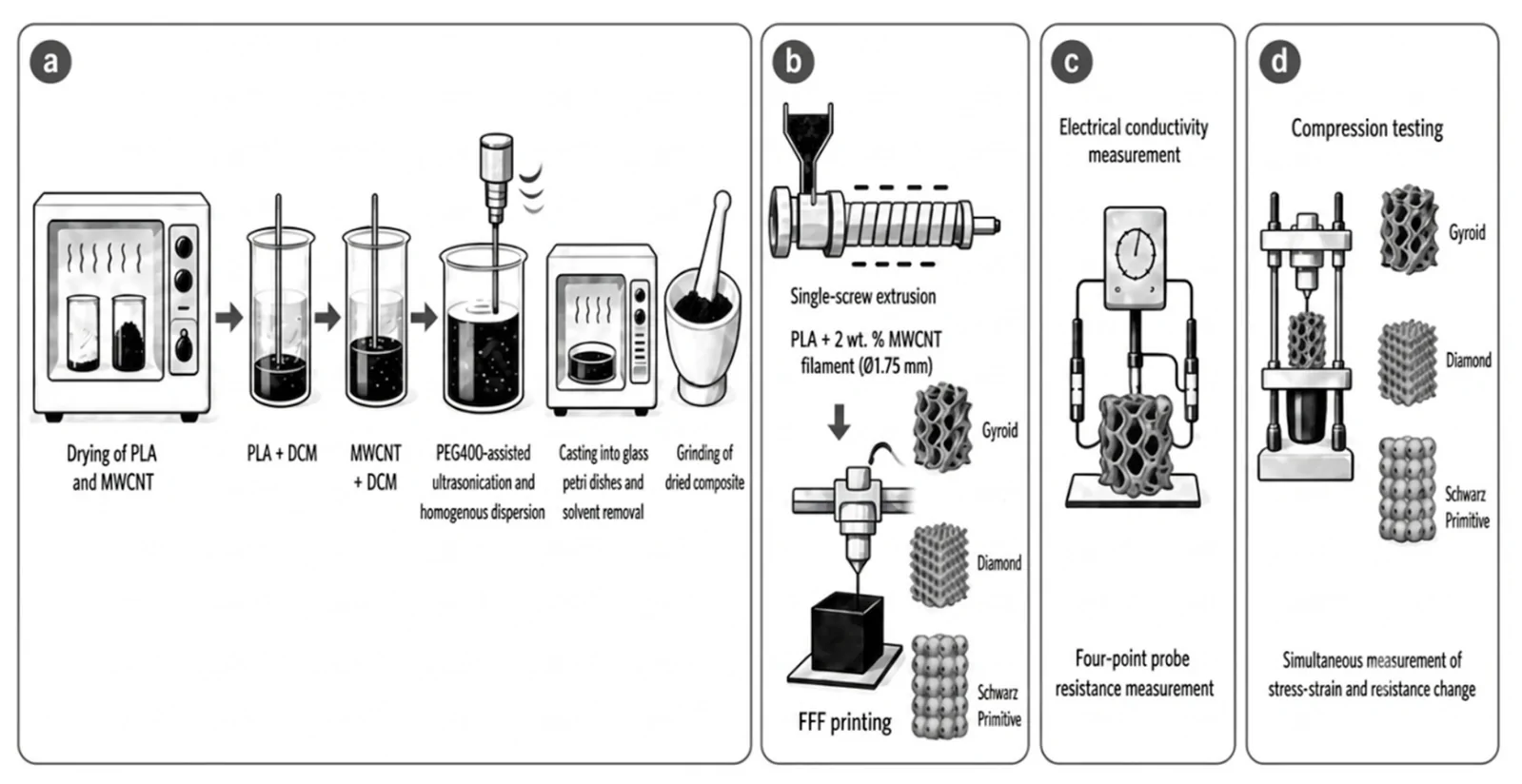
Schematic representation of the production and characterization of TPMS lattice structures with composite filaments: (**a**) Production process of MWCNT-reinforced PLA polymer via the solvent mixing method. (**b**) Composite filament production and the FFF-based production process of TPMS lattice structures. (**c**) Electrical conductivity measurement process of TPMS lattice structures. (**d**) Investigation process of the piezoresistive behavior of TPMS lattice structures.

Accordingly, in order to produce 100 g of conductive filament (2% MWCNT-reinforced PLA filament) using the solution mixing method schematically shown in Figure 1a, first 97.4 g of PLA granules (PLA granules; 3devo B.V., Utrecht, The Netherlands) and 2 g of MWCNT (MWCNTs; Nanografi, Ankara, Turkey) were dried in an oven at 85 °C for 12 h, and then the PLA granules were dissolved in dichloromethane (DCM) (DCM; Merck KGaA, Darmstadt, Germany) by stirring with a mechanical stirrer for 30 min. Subsequently, 2 g of MWCNT and 0.6 g of PEG400 dispersion agent (PEG 400; Merck KGaA, Darmstadt, Germany) were mixed in DCM using an ultrasonicator (Ultrasonicatör; Hielscher Ultrasonics GmbH, Teltow, Germany) for 15 min. Finally, both mixtures were mixed again using an ultrasonicator for 30 min to prevent agglomeration and ensure homogeneity. The resulting mixture was dried in an oven at 55 °C for 6 h to promote the evaporation of DCM, which has a boiling point of approximately 39.6 °C. After drying, the MWCNT-reinforced PLA material was mechanically ground and converted into granules. Any residual volatile solvent was further removed during the subsequent melt-extrusion process, which was conducted at 170–185 °C, and during FFF printing at 220 °C.

### 2.2. Filament Extrusion

A filament with a diameter of 1.75 mm was produced from the manufactured MWCNT-reinforced PLA granules using a single-screw extruder (Model SJ35, Changzhou Qubang Machinery Co., Ltd., Qubang, China), as shown in Figure 1b. The extruder screw speed set for filament production was 40 rpm, while the extruder temperatures were 170 °C for the feeding temperature, 185 °C for the extruder temperature, and 180 °C for the nozzle temperature, respectively.

Figure 1 was generated with the assistance of an AI tool (Abacus.AI/OpenAI GPT-4o Image Generation) to schematically illustrate the multi-step experimental workflow, including (a) solvent-assisted preparation of MWCNT/PLA granules, (b) single-screw composite filament extrusion and FFF 3D printing of TPMS lattice architectures, (c) four-point probe electrical conductivity measurement, and (d) in-situ compression piezoresistive testing. The prompt used for generating the schematic illustration was: “Create a professional, academic scientific workflow diagram for a polymers journal: (a) solvent mixing of PLA pellets and multi-walled carbon nanotubes (MWCNTs) with dichloromethane and ultrasonication, (b) filament extrusion (1.75 mm) and FFF 3D printing of TPMS lattice structures (Gyroid, Diamond, Schwarz Primitive), (c) four-point probe electrical conductivity testing setup with silver paste contacts, and (d) electromechanical compression test setup with digital multimeter recording resistance change under load. Clean vector aesthetic, white background, scientific color palette.” The authors thoroughly reviewed, verified, and adapted all visual elements to ensure scientific accuracy and take full responsibility for the integrity and content of this publication.

### 2.3. Creation of TPMS Lattice Structures

In this study, TPMS lattice structures with Gyroid (G), Diamond (D), and Schwarz Primitive (P) topologies were created using computer-aided design (CAD) (nTopology (nTop, New York, NY, USA)) software. TPMS mid-surfaces were defined using implicit surface equations. The basic TPMS equations used in this study are given in Table 2. The unit cell size was kept constant for all topologies and selected as α = 4 mm. Thus, each specimen was evaluated geometrically at the same cell scale, and effects that could arise from cell size in the comparison between topologies were minimized. The external dimensions of the lattice structures were determined as 16 × 16 × 24 mm^3^ for all topologies, and this sizing was chosen to ensure a 4 × 4 × 6 full cell array with a 4 mm unit cell size, thereby reducing partial cell effects that could occur in boundary regions and supporting representative structural behavior [28,29]. Because the specimens were porous cellular metamaterials rather than solid bulk polymers, ISO 13314 [30] was utilized as the primary testing standard to govern the compression characterization of the cellular structures, whereas the principles of ASTM D695-15 [31] served only as a secondary reference regarding the height-to-width aspect ratio (1.5:1) and crosshead alignment. In accordance with ISO 13314 guidelines for cellular solids, nominal apparent dimensions were used for macroscopic stress and strain evaluations, and uniaxial compression was executed at a constant crosshead speed of 3 mm/min.

In order to reveal the variation in piezoresistive behavior solely depending on topology, the relative density (RD) value was kept constant in all lattice structures, and the target RD = 0.30 (30%) was selected for all topologies. To ensure the constant RD condition, the surface thickness parameter was adjusted separately for different TPMS topologies. The variation in thickness values from one topology to another results from the differences in the specific surface area, curvature distribution, and volumetric filling characteristics of TPMS geometries [32]. Within this scope, the surface thicknesses determined for TPMS lattice structures with Gyroid, Diamond, and Schwarz Primitive topologies, as well as their total volumes and solid volumes, are shown in Table 2. This approach enabled the piezoresistive behaviors of different TPMS architectures to be investigated under the same boundary conditions, with an equivalent material ratio, and in a directly comparable manner. The external volume of each specimen was 6.144 cm^3^, based on the dimensions of 16 × 16 × 24 mm^3^. The measured average masses were 2.95 g for Gyroid, 3.40 g for Diamond, and 2.48 g for Schwarz Primitive. Accordingly, the measured apparent mass densities were 0.480, 0.553, and 0.404 g cm^−3^, respectively. Using the PLA density given in Table 1 (1.24 g cm^−3^) as the reference solid-material density, the corresponding experimental relative densities were calculated as 38.72% for Gyroid, 44.63% for Diamond, and 32.55% for Schwarz Primitive. Compared with the nominal CAD relative density of 30%, the deviations were +29.07%, +48.76%, and +8.51%, respectively. These results indicate that the printed structures did not have exactly the same relative density, and therefore the measured relative-density differences should be considered when comparing their mechanical and electrical responses.

### 2.4. Production of TPMS Lattice Structures Using the FFF Method

The TPMS lattice structures in this study were produced in XY orientation using FFF, one of the AM methods, as specified in the schematic representation in Figure 1b. For this purpose, a Creality model (K1C, Creality Co., Ltd., Shenzhen, China), capable of printing 1.75 mm diameter carbon-based reinforced polymer, was used. The processing parameters used to produce the TPMS lattice structures are given in Table 3.

To ensure that the observed mechanical and electrical differences were governed strictly by intrinsic cell topology rather than manufacturing defects or variations in printing quality, all topologies were printed using identical slicing configurations, nozzle kinematics, and thermal parameters without support structures. Due to the continuous minimal surface nature of TPMS geometries, overhang angles were naturally self-supporting, avoiding local sagging or discontinuous bead deposition. Furthermore, dimensional fidelity and mass consistency across manufactured specimens were verified prior to testing; the recorded specimen masses (Table 4) remained within close tolerance of the theoretical solid volume designs (Table 2), demonstrating consistent material deposition and minimal printing-induced porosity among the different topologies. For each TPMS topology (Gyroid, Diamond, and Schwarz Primitive), three identical independent replicate specimens were successfully fabricated (*n* = 3 per group, yielding a total of 9 specimens) to ensure statistical reliability and experimental repeatability across all subsequent mechanical, electrical, and piezoresistive characterizations. In FFF processing, print resolution (governed by the 0.2 mm layer height and 0.4 mm nozzle diameter) and the layer deposition direction directly dictate the mesostructural characteristics of the specimens. All TPMS specimens were built vertically along the Z-axis, meaning that individual filament beads were deposited in the horizontal XY planes while compressive mechanical loading was subsequently applied parallel to the Z-axis (normal to the inter-layer weld interfaces). Because TPMS architectures feature smoothly varying spatial curvatures, the interaction between layer thickness and local surface slope inherently introduces topological staircase effects (stair-stepping), generating characteristic micro-scale surface roughness along inclined cell boundaries.

### 2.5. Characterization Analyses

#### 2.5.1. Mechanical Performance

Hardness and compression tests were performed to evaluate the mechanical properties of MWCNT-reinforced PLA composites produced with FFF. Hardness values were measured using a Shore D hardness tester (LXD-M01D, Loyka, Mumbai, Turkey), in accordance with the ASTM D2240 [33] standard. Compression tests were performed at room temperature (23 ± 2 °C) using a DEVOTRANS universal testing machine (DVT 20KN, DEVOTRANS, Istanbul, Turkey) equipped with a 20 kN load cell at a constant crosshead speed of 3 mm/min until 50% compressive strain was reached. Three independent specimens (*n* = 3) were tested for each TPMS topology under identical conditions. Because the investigated specimens were porous TPMS lattice structures rather than solid bulk polymers, the testing protocol was adapted to the cellular architecture: ISO 13314 was adopted as the primary governing standard for the mechanical testing and evaluation of porous and cellular geometries, while ASTM D695-15 was employed strictly as secondary guidance for general specimen aspect ratio and test platen alignment. The compressive engineering stress and nominal strain were calculated using the apparent (external) specimen dimensions rather than the locally varying internal solid cross-sectional area. Specifically, compressive stress was calculated by dividing the instantaneous compressive force by the full initial external (apparent) cross-sectional area of the specimen (16 mm × 16 mm = 256 mm^2^) rather than the projected solid area or the locally varying internal solid cross-section, in strict accordance with ISO 13314 guidelines for cellular materials. Similarly, compressive engineering strain was obtained by dividing the crosshead displacement by the initial external height of the specimen (24 mm). This apparent stress–strain approach was deliberately adopted in accordance with cellular solids testing conventions to enable direct, standardized performance comparisons across the different TPMS topologies. The compressive stress at 50% strain and specific energy absorption (SEA) values were evaluated from the resulting stress–strain data and are reported as mean ± standard deviation. All mechanical results were obtained from three independent specimens per topology and are reported as mean ± standard deviation; statistical comparisons among the TPMS topologies were performed using one-way ANOVA followed by Tukey’s HSD post-hoc test, as described in Section 2.5.7. Specifically, the total energy absorption per unit volume (W) was calculated by integrating the area under the compressive stress–strain curve up to 50% compressive strain (*ε* = 0.50), and the specific energy absorption (SEA, J g^−1^) was determined by normalizing the absorbed energy by the actual mass of the tested specimen, according to Equation (1):

(1) $$\mathrm{SEA}=\frac{v_{app}}{m}\int_{0}^{\varepsilon_{max}}\sigma \left(\varepsilon \right)d\varepsilon$$

where *σ(ε)* is the instantaneous compressive engineering stress, *ε* is the compressive strain, *ε_max_* is the maximum evaluation strain (*ε_max_ =* 0.50), *v_app_* is the apparent external volume of the specimen (6144 mm^3^) and *m* is the measured specimen mass in grams.

#### 2.5.2. Materials’ Spectroscopic Characterization

The filament produced within the scope of this study and the polymer structure using MWCNT were primarily analyzed using FTIR analyses. A Perkin-Elmer Spectrum Two model FTIR spectrophotometer (Spectrum Two, PerkinElmer, Waltham, MA, USA) was used for these analyses. FTIR analyses were performed between 400 cm^−1^ and 4000 cm^−1^ with a measurement sensitivity of 4 cm^−1^. FTIR measurements were carried out using an ATR module.

#### 2.5.3. Materials’ Thermal Characterization

Thermal analyses (DTA, TGA, DSC) were performed for MWCNT, PLA, and composite structures in the study [34]. The thermal stability of the structures obtained in these analyses was determined by Thermo-Gravimetric Analysis (TGA). The degradation onset temperatures were determined by Differential Thermal Analysis (DTA), and the glass transition temperatures were determined by Differential Scanning Calorimetry (DSC). A Shimadzu TGA-50 model thermo gravimeter (TGA-50, Shimadzu Corporation, Kyoto, Japan)was used for the thermogravimetric analyses. The TGA runs were performed from 20 °C to 700 °C at a constant heating rate of 10 °C/min under a static air (oxidative) atmosphere without purge gas. This thermo-oxidative environment was deliberately chosen to assess both the oxidative thermal degradation of the PLA matrix and the oxidation resistance of the MWCNT filler, as evidenced by the high-temperature carbon oxidation observed above 500 °C. DTA analyses were carried out between 10 °C and 550 °C using a Shimadzu DTA-50 model device (DTA-50, Shimadzu Corporation, Kyoto, Japan) at a heating rate of 10 °C/min. The analyses were performed with 10 mg of sample and reference material under a static air atmosphere. Alpha-alumina was used as the reference material in the analysis. DSC analyses were carried out under an air atmosphere using a Shimadzu DSC-60 model device (Shimadzu Corporation, Kyoto, Japan ) at a heating rate of 10 °C/min. In these analyses, alpha-alumina was used as the reference material, and indium was used for device calibration.

#### 2.5.4. Scanning Electron Microscopy (SEM)

Microstructural and morphological analyses of MWCNT-reinforced PLA composites were performed using a LEO-EVO 40 scanning electron microscope (SEM) (LEO-EVO 40, Carl Zeiss SMT, Oberkochen, Germany). The fracture surfaces were coated with a thin gold–palladium (Au-Pd) layer using a BAL-TEC SCD 050 coating device (SCD 050, BAL-TEC AG, Balzers, Liechtenstein) to ensure the electrical conductivity of the polymeric structure and prevent charging during imaging. To obtain high-resolution images from the specimens, an acceleration voltage of 20 kV–40 kV and a secondary electron (SE) detector (LEO-EVO 40, Carl Zeiss SMT, Oberkochen, Germany) were used under high-vacuum conditions.

#### 2.5.5. Electrical Conductivity Measurement

Electrical conductivity measurements were performed using the four-probe method, as schematically shown in Figure 1c. The electrical measurements of the specimens were carried out using a Tektronix DMM4050 digital multimeter (DMM4050, Tektronix, Beaverton, OR, USA) with 6.5-digit high-resolution sensitivity [35]. To ensure statistical reliability and quantify measurement dispersion, three independent specimens (*n* = 3) were tested for each TPMS topology under identical ambient conditions (23 ± 2 °C, 50 ± 5% RH), and baseline electrical conductivity values are reported as mean ± standard deviation. To ensure rigorous electrical interfacing, high-conductivity colloidal silver paint was applied to the specimen contact boundaries and allowed to fully cure at ambient temperature prior to any electrical characterization. Consequently, all baseline (initial) electrical conductivity measurements were conducted strictly after the application and complete drying of the silver conductive layer. To resolve the issue of contact resistance on these complex cellular architectures, the four-probe (Kelvin) sensing configuration was deliberately employed. In this setup, an excitation current is injected through the outer electrode pair while the potential drop is measured across the inner high-impedance voltage probes. Because the internal input impedance of the Tektronix DMM4050 digital multimeter is extremely high (>10 GΩ), the current flowing through the voltage-sensing circuit is virtually zero (I ≈ 0) thereby intrinsically eliminating the parasitic contact resistances (R_c_) between the probes and the porous composite surface, as well as lead resistances. Furthermore, the cured silver paint layer serves as an equipotential boundary pad across the discrete solid struts, ensuring uniform macroscopic current distribution into the complex TPMS lattice networks without localized current crowding. For highly non-uniform cellular architectures such as TPMS lattice structures, the solid cross-sectional area continuously varies along the conduction path. Therefore, following established conventions for conductive cellular solids and porous composites, an apparent (macroscopic) bulk conductivity (*σ*) was calculated using the apparent external dimensions of the specimens rather than the local solid cross-section, according to Equation (2):

(2) $$\sigma =\frac{L}{RA}$$

where *R* is the measured electrical resistance, *L* is the effective conduction length along the loading direction (*L* = 24 mm), and *A* is the apparent macroscopic cross-sectional area perpendicular to the current flow (16 mm × 16 mm = 256 mm^2^). Because all TPMS topologies were designed with identical external boundary dimensions (16 × 16 × 24 mm^3^) and constant relative density (30%), this apparent conductivity formulation provides a direct and standardized basis for comparing the topology-dependent electron-transport pathways across the Gyroid, Diamond, and Schwarz Primitive architectures.

#### 2.5.6. Piezoresistive Behavior

The piezoresistive behaviors of TPMS lattice structures produced by the FFF method were measured under compressive loading, as schematically illustrated in Figure 1d. Compression tests were conducted using a DEVOTRANS universal testing machine with a capacity of 20 kN, while electrical resistance changes were recorded in-situ using a 6.5-digit high-resolution Tektronix DMM4050 digital multimeter. The metallic compression platens were electrically insulated using non-conductive polyimide (Kapton) tape to isolate the test specimen from the machine frame. To characterize the electromechanical response along the primary deformation axis, the four-probe electrodes were configured parallel to the uniaxial loading direction (along the 24 mm height). Specifically, current-injecting terminals and inner voltage-sensing leads were connected to the opposing top and bottom boundary surfaces through cured high-conductivity colloidal silver paint layers. To maintain stable, uninterrupted electrical contact throughout extensive progressive deformation (up to 50% strain) without introducing mechanical constraint, ultra-flexible, multi-strand copper lead wires were embedded directly into the silver contact pads. The axial compressive force exerted by the platens continuously ensured intimate, void-free interfacial contact between the contact faces and the electrodes, while the mechanical compliance of the flexible leads successfully accommodated crosshead displacement without inducing interfacial peeling, localized stress concentrations, or parasitic probe detachment. Simultaneous electromechanical compression tests were conducted on three independent specimens per architecture (*n* = 3) to confirm the repeatability of the piezoresistive response. To quantify the electromechanical sensitivity across the nonlinear deformation range (10–50% compressive strain), the Gauge Factor (GF) was calculated. Because porous lattice structures undergo progressive cell deformation and nonlinear resistance changes over large compressive strain regimes, the secant gauge factor at specific strain thresholds (10%, 25%, and 50%) was determined using Equation (3):

(3) $$GF=\frac{\left|\frac{\Delta R}{R_{0}}\right|}{\varepsilon }$$

where *R*_0_ is the initial electrical resistance prior to mechanical loading, *R* is the instantaneous electrical resistance at the designated compressive strain, *ΔR = R − R*_0_ represents the absolute resistance change, and ε is the applied apparent compressive strain. Evaluating the secant GF at distinct strain intervals allows for a direct and robust comparative assessment of topology-dependent sensitivity during initial elasticity (10%), progressive cell collapse (25%), and extensive densification (50%).

#### 2.5.7. Statistical Analysis

All experimental quantitative data (specimen mass, compressive stress at 50% strain, specific energy absorption, Shore D hardness, and initial electrical conductivity) are expressed as mean ± standard deviation for three independent replicates (*n* = 3). One-way analysis of variance (ANOVA) followed by Tukey’s honestly significant difference (HSD) post-hoc test was performed to evaluate the statistical significance of differences between the three TPMS topologies (Diamond, Gyroid, and Schwarz Primitive). A threshold value of *p* < 0.05 was established as the criterion for statistical significance (* *p* < 0.05, ** *p* < 0.01, and *** *p* < 0.001).

## 3. Results and Discussion

### 3.1. Mechanical Properties

Hardness measurements, compression tests conducted to determine mechanical properties, specimen masses, and SEA values are given in Table 4. Accordingly, the TPMS cell topology significantly affected the mechanical performance of the lattice structures. The Diamond topology exhibited the highest compression stress (14.164 ± 0.164 MPa), specific energy absorption capacity (9.161 ± 0.191 J g^−1^), and Shore D hardness value (75.6 ± 0.2). This situation can be attributed to Diamond geometry providing a more effective load transfer under load and absorbing deformation energy more efficiently. The Gyroid structure showed an intermediate performance in terms of compression stress (8.777 ± 0.327 MPa) and SEA value (6.149 ± 0.199 J g^−1^). In contrast, the Schwarz Primitive topology had the lowest compression stress (3.152 ± 0.202 MPa), SEA (2.917 ± 0.147 J g^−1^), and hardness (71.2 ± 2.2) values; thus, it provided the lowest performance in terms of mechanical load bearing and energy absorption among the investigated topologies. One-way ANOVA confirmed that the TPMS unit-cell topology exerted a statistically significant influence on compressive stress (F = 1562.67, *p* < 0.001), specific energy absorption (F = 898.32, *p* < 0.001), and surface hardness (F = 8.50, *p* = 0.018). Post-hoc pairwise comparisons verified that the differences in both compressive stress and SEA between all topological pairs (Diamond vs. Gyroid, Diamond vs. Primitive, and Gyroid vs. Primitive) were statistically significant (*p* < 0.001). In this context, the Diamond topology stands out as the most suitable structure for applications requiring mechanical strength and energy absorption.

#### 3.1.1. Hardness Test Results

Shore D hardness measurements were carried out to determine the initial surface hardness of the specimens before they were subjected to the compression test. Therefore, the obtained values reflect the initial surface resistance of the produced TPMS structures, independent of the deformation or damage occurring during compression loading. According to the data presented in Table 4, the Diamond topology reached the highest initial hardness value with 75.6 Shore D. The initial hardnesses of the Gyroid and Schwarz Primitive structures were measured as 73.5 and 71.2 Shore D, respectively. The limited difference between the topologies can be attributed to the fact that all specimens were produced using the same MWCNT-reinforced PLA filament, FDM production parameters, and production orientation. However, the higher initial hardness of the Diamond topology is consistent with the high compression stress and specific energy absorption values obtained in the compression test.

#### 3.1.2. Compression Test Results

**Figure 2:**
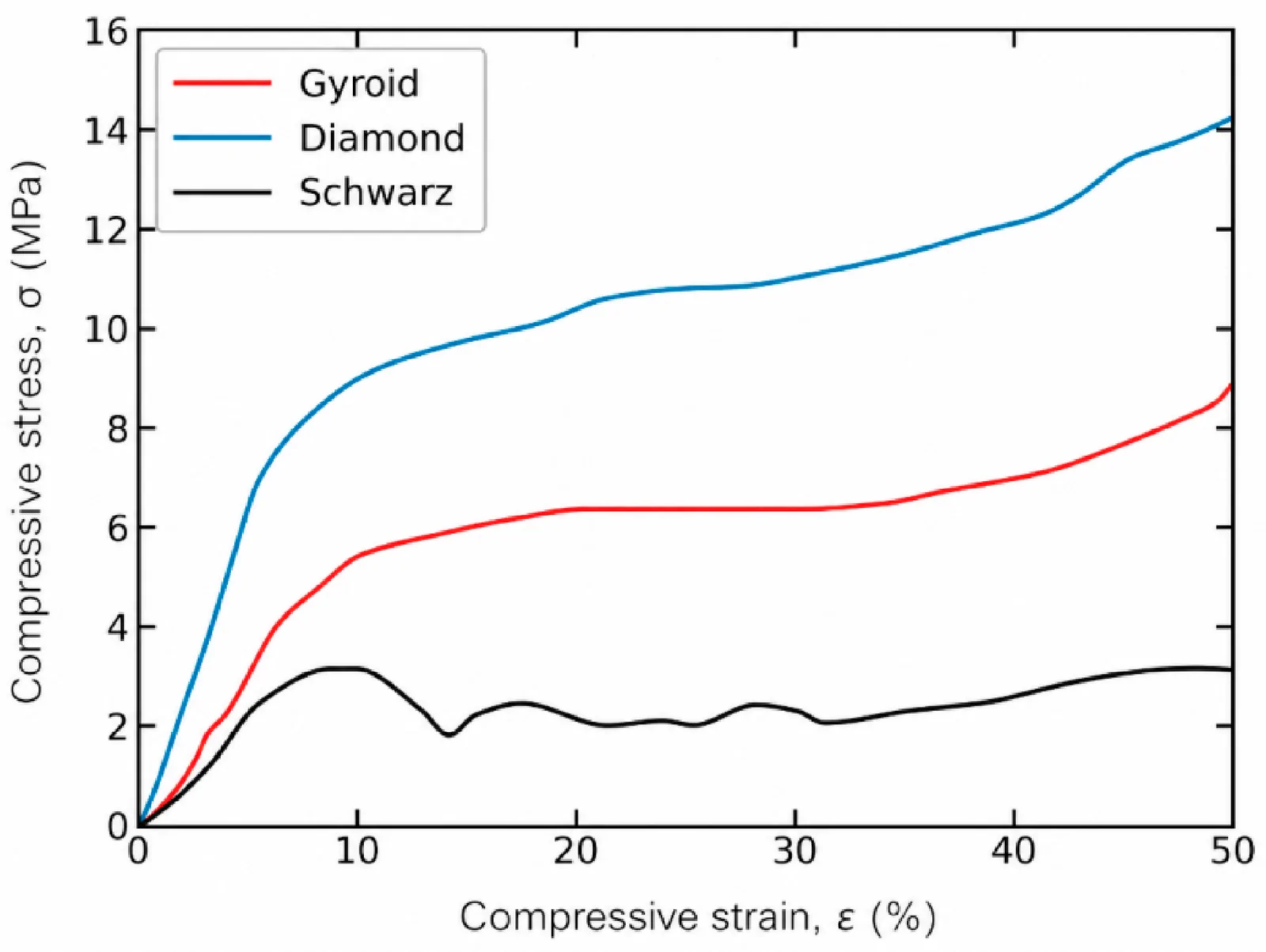
Stress–strain curves of TPMS lattice structures.

The compressive stress–strain curves given in Figure 2 show that TPMS cell topology significantly affects the deformation mechanism and load-bearing capacity under loading. Although an initially increasing stress trend was observed in the low-strain region for all specimens, the behavior of the curves at subsequent stages differed depending on the topology. The Diamond topology exhibited the highest compressive stress throughout the entire strain range. The rapid increase in stress within the 5–10% strain range indicates that this topology has high initial stiffness. Although a limited plateau region was observed after approximately 20% strain, the stress value generally continued to increase and reached approximately 14.164 MPa at 50% strain. This continuous increase shows that the Diamond structure provides more stable load transfer under compressive loading and that its densification tendency is more pronounced than that of the other topologies. The Gyroid structure exhibited lower compressive stress than the Diamond topology but higher compressive stress than the Schwarz Primitive structure. During the initial deformation stage, the stress increased rapidly; however, a relatively stable plateau region at approximately 6 MPa was formed within the 10–30% strain range. This behavior indicates that the Gyroid structure exhibits a controlled and more homogeneous deformation mechanism. After 30% strain, the stress increased again, and the structure reached a compressive stress of approximately 8.777 MPa at 50% strain. The Schwarz Primitive topology exhibited the lowest compressive stress values. An initial peak value of approximately 3.152 MPa was observed in the curve at approximately 5–10% strain, followed by a significant decrease in stress. This decrease can be associated with local buckling in the cell walls, layer-based collapse, or the onset of early damage. The fluctuating curve behavior within the approximately 10–30% strain range indicates that the structure exhibited progressive collapse behavior in successive cell rows. The stress remaining at approximately 3.1 MPa at 50% strain reveals that the Schwarz Primitive topology has the lowest load-bearing capacity among the investigated structures. The observed mechanical responses and failure modes are strongly correlated with the interplay between FFF build characteristics (print resolution, layer lay direction, and surface roughness) and the geometric nature of each topology. Because uniaxial compression was applied parallel to the Z-axis (normal to the inter-layer deposition planes), the structural struts were subjected to out-of-plane compressive and shear stresses. For the Diamond topology, the continuous spatial inclination and dual-strut network effectively distribute the compressive load across multiple intersecting angles, where compressive forces act to close inter-layer weld gaps and suppress inter-laminar delamination, resulting in high stiffness and delayed yielding. In contrast, for the Schwarz Primitive topology, the presence of vertical flat wall regions and sharp transition radii aligns the local inter-layer weld interfaces directly with peak out-of-plane shear stress trajectories. Consequently, stair-stepping ridges and local surface roughness act as geometric notch-like stress concentrators, promoting premature inter-layer shear cleavage, localized wall buckling, and the pronounced post-yield stress drops observed in Figure 2. For the Gyroid topology, its uniform zero-mean curvature minimizes localized stress concentrations along the stepped layer boundaries, enabling smooth stress transfer and a stable, ductile-like plateau behavior. Thus, while identical printing parameters (0.2 mm layer height, XY raster lay) were applied to all groups, the intrinsic topology dictates how manufacturing-induced surface roughness and layer-boundary orientations correlate with macroscopic load bearing.

#### 3.1.3. Specific Energy Absorption

**Figure 3:**
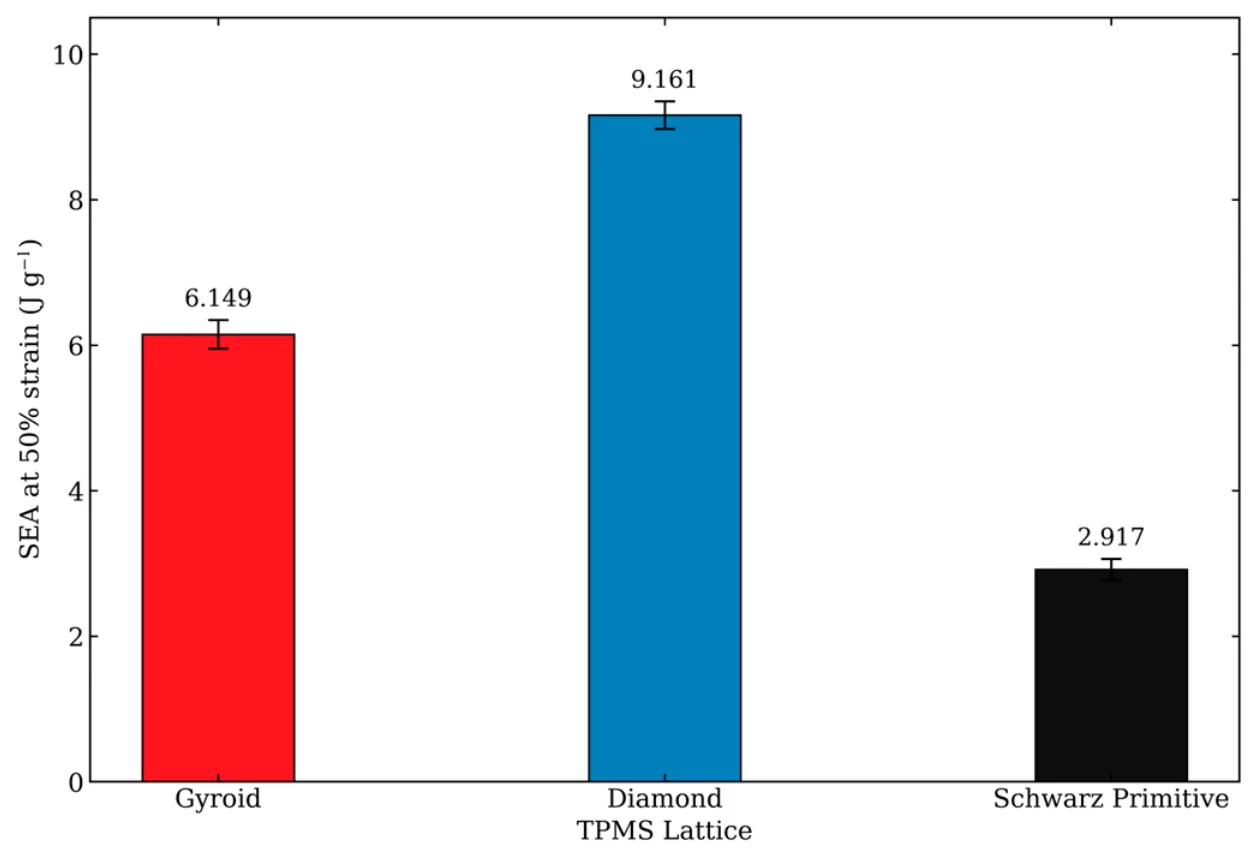
Specific Energy Absorption (SEA) of TPMS Lattice Structures.

The specific energy absorption (SEA) values given in Figure 3 show that TPMS topology has a significant effect on the energy absorption capacity per unit mass. At 50% strain level, the highest SEA value was obtained in the Diamond topology as 9.161 J g^−1^. This value indicates that the Diamond structure absorbs energy more effectively under compressive loading compared to the Gyroid and Schwarz Primitive topologies. The Gyroid structure exhibited a moderate energy absorption performance with a SEA value of 6.149 J g^−1^. Although the continuous surface geometry of the Gyroid topology provided a more balanced deformation behavior during compression, the energy absorbed per unit mass remained at approximately 65% of the Diamond structure. In contrast, the Schwarz Primitive topology showed the lowest SEA value with 2.917 J g^−1^. This result is consistent with the early stress drops and progressive cell collapse behavior observed in the compressive stress–strain curve. The low load-bearing capacity of the Primitive structure caused the area under the curve up to 50% strain, and therefore the energy absorption capacity, to remain limited.

### 3.2. Fourier Transform Infrared Spectroscopy (FTIR)

**Figure 4:**
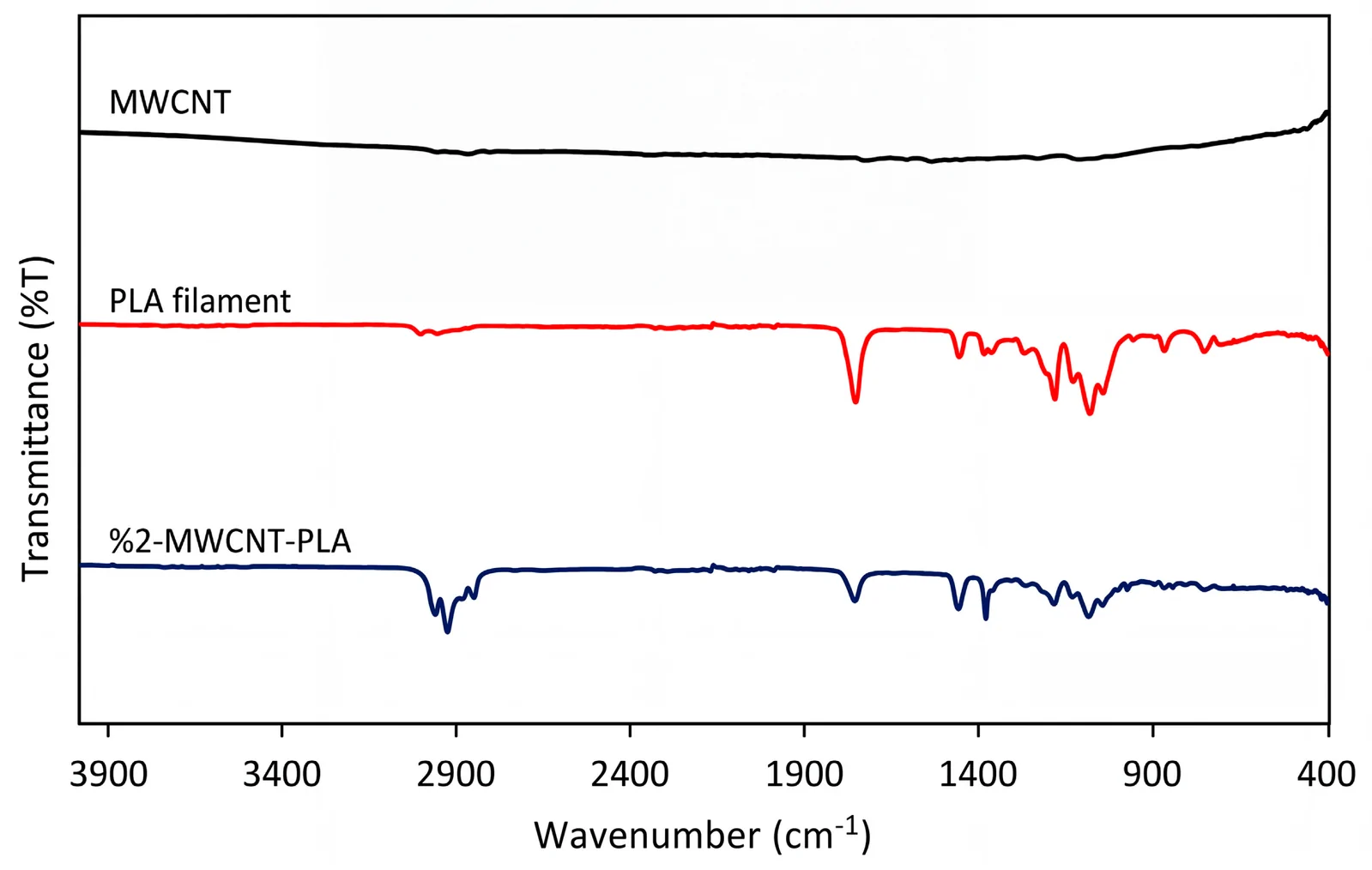
FTIR analysis of MWCNT, PLA, and MWCNT/PLA composites with 2 wt%.

FTIR spectra of pure PLA, MWCNT, and 2% MWCNT/PLA composite filament samples across the full spectral range (4000–400 cm^−1^) are presented in Figure 4. To facilitate a comprehensive comparative identification of the functional groups and vibrational modes, the experimental wavenumbers of key characteristic absorption bands alongside their chemical assignments are summarized in Table 5.

The MWCNT spectrum generally exhibits low absorption intensity and a predominantly flat baseline, which is typical for pristine, unfunctionalized carbon nanotubes possessing limited IR-active dipolar functional groups. In this spectrum, a weak band at approximately 2918 cm^−1^ corresponds to C–H stretching, while the subtle band around 1530 cm^−1^ is assigned to the skeletal vibration of the graphitic carbon backbone (aromatic C=C stretching). In contrast, characteristic vibrational fingerprints of the polyester backbone dominate the pure PLA spectrum. The prominent, sharp absorption peak observed at 1748 cm^−1^ corresponds to the stretching vibration of the ester carbonyl group (C=O). The asymmetric and symmetric deformation vibrations of the methyl (–CH_3_) groups are resolved at 1452 cm^−1^ and 1360 cm^−1^, respectively. The complex spectral envelope spanning 1180–1080 cm^−1^ is attributed to the stretching vibrations of ester C–O–C bonds, accompanied by the C–CH_3_ stretching mode at 1044 cm^−1^ and skeletal ester chain vibrations at 868 cm^−1^ and 754 cm^−1^. The low-intensity doublet in the 2995–2946 cm^−1^ range corresponds to asymmetric and symmetric aliphatic C–H stretching vibrations of the polymer main chain. The FTIR spectrum of the 2 wt.% MWCNT/PLA composite reveals that PLA essentially retains all its primary characteristic absorption bands, and no new absorption bands or distinct chemical peak shifts occurred during solvent processing and melt compounding. This preservation confirms that no covalent chemical bonding was formed between the unfunctionalized MWCNTs and the PLA matrix. Nevertheless, subtle spectral modifications are discernible: the ester carbonyl band at 1748 cm^−1^ shows slight broadening and intensity attenuation, while the aliphatic C–H stretching bands (2995–2946 cm^−1^) exhibit minor intensity variations. These phenomena reflect secondary physical interactions, including non-covalent interfacial van der Waals forces, subtle perturbations in polymer chain packing, and the nucleating influence of dispersed nanotubes on local matrix crystallization, rather than chemical degradation or functionalization. No additional absorption bands attributable to residual DCM were observed in the FTIR spectrum of the 2 wt.% MWCNT/PLA composite. This observation supports the effective removal of DCM during the drying and subsequent thermal-processing steps.

### 3.3. Thermal Characteristics

#### 3.3.1. Thermo-Gravimetric Analysis (TGA)

**Figure 5:**
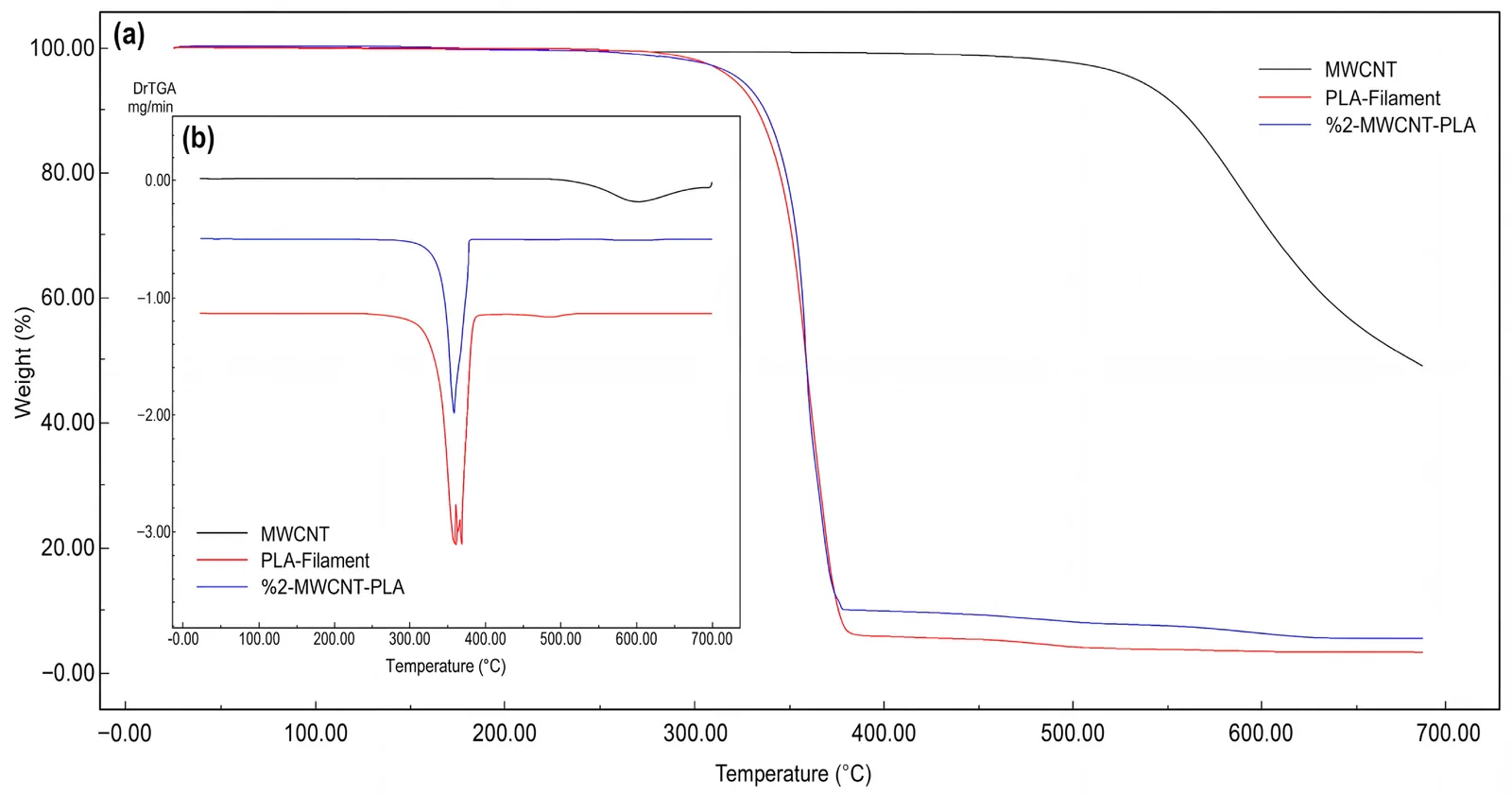
MWCNT, PLA, and 2 wt% MWCNT/PLA composite structures; (**a**) TGA thermograms, (**b**) differential TGA curves.

TGA thermograms of the composite structure and its components produced within the scope of the study are presented in Figure 5. Unlike the mechanical and electrical measurements, which were replicated across three independent specimens (*n* = 3) and reported as mean ± standard deviation, the TGA, DTA, and DSC analyses were performed as single representative runs per material, consistent with standard thermal-analysis practice, since thermogravimetric behavior of a given material composition shows negligible run-to-run variability under fixed instrumental conditions. Therefore, the curves presented in Figure 5 correspond to single representative measurements rather than averaged values, and error bars are not applicable to this figure. While no mass loss was observed in the thermogram of the MWCNT sample up to approximately 500 °C, a mass loss of approximately 50% occurred between 500 °C and 700 °C due to the oxidation of the carbon structure. When the thermogram of the pure PLA filament is examined, a sharp mass loss starts at approximately 300 °C and continues until 400 °C is observed. This situation shows that the material is thermally stable up to 300 °C, and from this temperature onwards, the polymer chains undergo thermal degradation. TGA data for the MWCNT/PLA composite structure revealed that thermal degradation started at approximately 340 °C. The increase in the degradation temperature of pure PLA from 300 °C to 340 °C in the composite structure proves that the addition of MWCNT increased the thermal stability of the material. Furthermore, the residual (ash) amount at 700 °C being measured as approximately 6% in the composite structure is a higher residue value compared to pure PLA and confirms the presence of MWCNT in the structure. These data obtained from the TGA confirm that composite filaments with the targeted properties and high thermal resistance were successfully produced.

#### 3.3.2. Differential Thermal Analysis (DTA)

**Figure 6:**
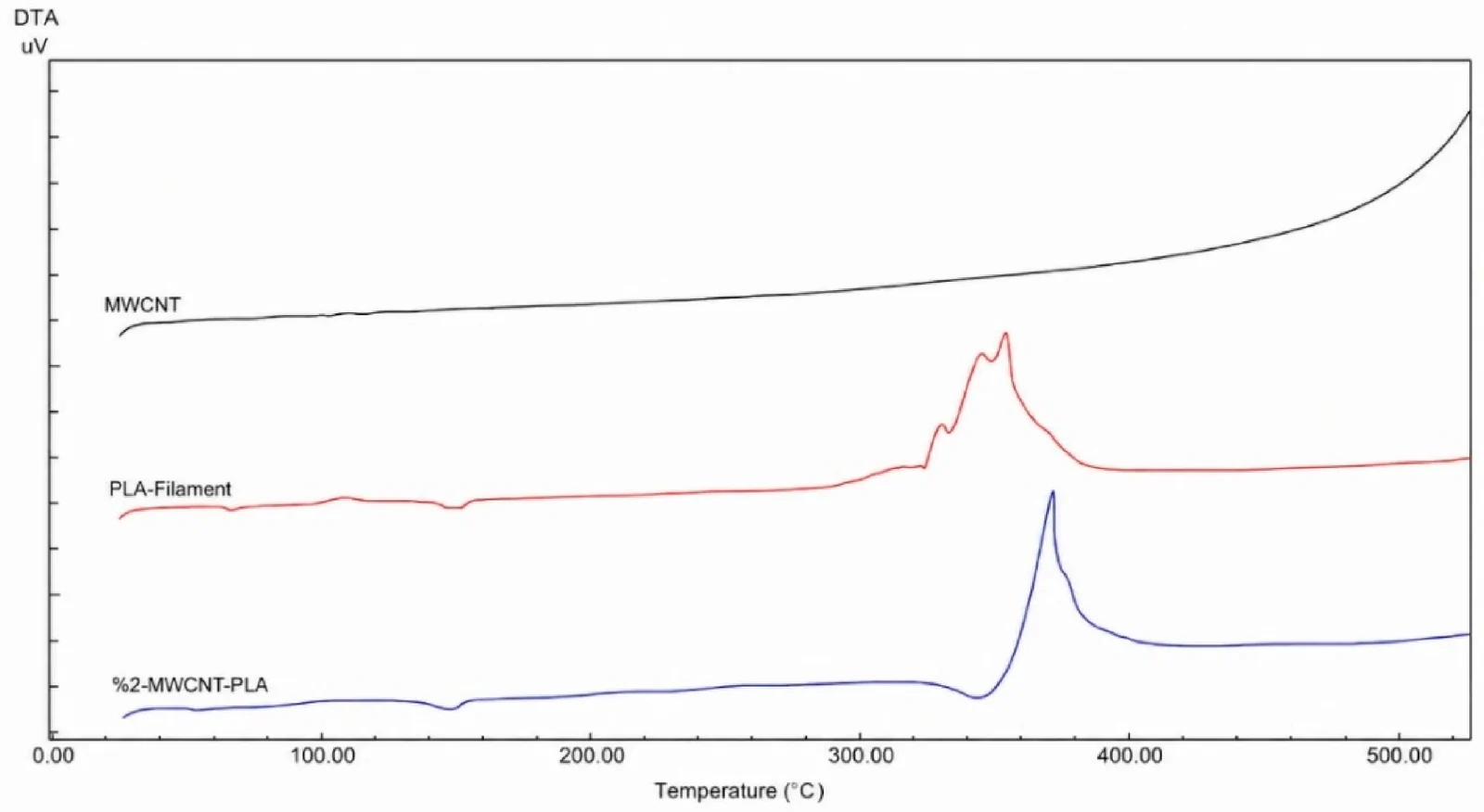
DTA curves of MWCNT, PLA, and 2 wt% MWCNT/PLA composite structures.

DTA curves of MWCNT, neat PLA, and 2 wt.% MWCNT/PLA composite structures are given in Figure 6. When these thermograms are examined, no thermal transition or decomposition peaks are observed for pristine MWCNTs up to 500 °C under static air. In contrast, prominent thermal degradation phenomena are clearly detected for neat PLA and composite structures. Neat PLA exhibits an exothermic thermal degradation profile initiating at approximately 300 °C and concluding around 390 °C, corresponding to the main-chain scission and thermo-oxidative decomposition of polyester segments. For the 2 wt.% MWCNT/PLA composite structure, the degradation peak shifts distinctly toward higher temperatures, starting at approximately 350 °C and terminating at approximately 450 °C. This substantial positive shift of ~50 °C in both onset and peak temperatures corroborates the TGA results, demonstrating that the well-dispersed MWCNTs effectively delay thermal degradation by acting as physical barriers against thermo-oxidative decomposition.

#### 3.3.3. Differential Scanning Calorimetry (DSC)

**Figure 7:**
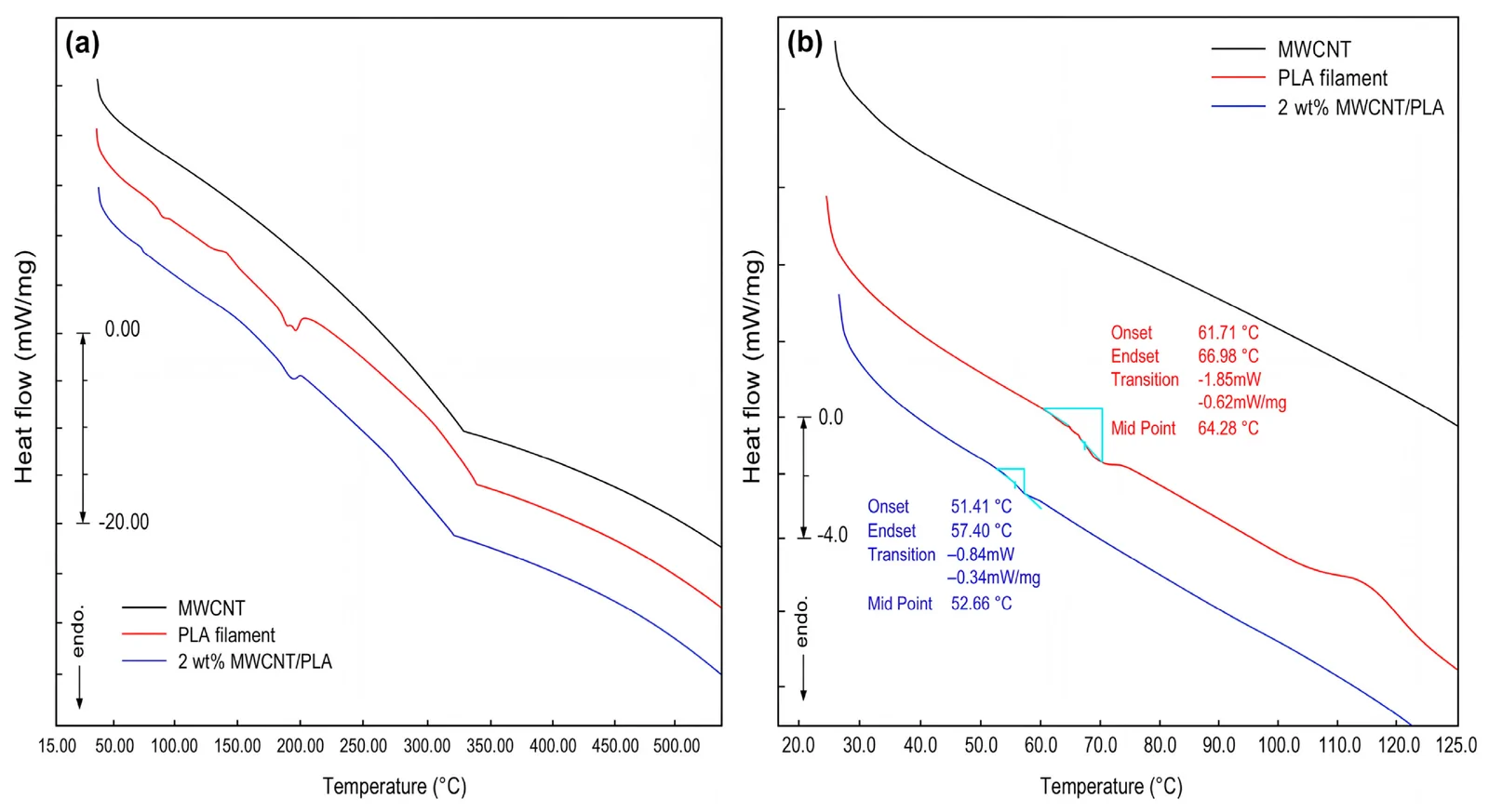
DSC analysis results of neat MWCNT, PLA filament, and 2 wt% MWCNT/PLA nanocomposite samples: (**a**) Full thermograms in the range of 15–500 °C. (**b**) Enlarged glass transition (Tg) region including onset, offset, and midpoint temperature values.

The thermal transition behaviors of MWCNT, pure PLA filament, and 2% MWCNT/PLA composite filament samples were investigated by differential scanning calorimetry (DSC) analysis in the temperature range of 15–500 °C. The general DSC curves obtained are given in Figure 7a, and the detailed view of the glass transition region is given in Figure 7b. No distinct glass transition temperature (Tg) was observed in the MWCNT sample within the investigated temperature range. This result is consistent with the fact that MWCNT is not a polymeric structure and does not exhibit glass transition behavior specific to amorphous polymers. In contrast, distinct glass transition regions were detected in pure PLA and 2% MWCNT/PLA composite filament samples. The onset, end set, and midpoint values of the glass transition temperature for pure PLA filament were determined as 61.71 °C, 66.98 °C, and 64.28 °C, respectively. In the 2% MWCNT/PLA composite, these values were measured as 51.41 °C, 57.40 °C, and 52.66 °C, respectively. Accordingly, the midpoint Tg exhibited an apparent decrease of approximately 11.62 °C (from 64.28 °C to 52.66 °C) upon composite fabrication. It is essential to reconcile this reduction in Tg with the concurrent enhancement in thermal degradation stability observed via TGA, as these two phenomena are governed by fundamentally distinct thermodynamic and physical mechanisms operating at widely disparate temperature regimes:

1. Thermal degradation stability (TGA, ~300–400 °C): The elevation in thermal decomposition temperature reflects the delay of covalent chain scission. Multi-walled carbon nanotubes act as efficient thermal dissipation networks and superior physical mass-transport barriers, which impede the volatilization and out-diffusion of primary pyrolysis degradation products from the bulk matrix.
2. Glass transition dynamics (DSC, ~50–65 °C): In contrast to thermal degradation, the glass transition temperature (Tg) is exclusively governed by the cooperative segmental mobility of amorphous PLA macromolecular chains and the evolution of polymer free volume. The marked depression in the midpoint Tg (from 64.28 °C to 52.66 °C) can be explained by two synergistic physicochemical mechanisms:

First, low-molecular-weight Polyethylene Glycol (PEG 400) was utilized during the solution-mixing stage as a surfactant/dispersing agent to assist MWCNT debundling. Residual oligomeric PEG chains effectively intercalate between adjacent PLA backbones, shielding intermolecular dipole–dipole interactions between the ester carbonyl groups. This acts as an internal plasticizer, lowering the energy barrier for conformational chain rotations and substantially increasing the macromolecular free volume.

Second, as corroborated by the FTIR findings, unfunctionalized (pristine) MWCNTs interact with the surrounding PLA chains strictly through weak, non-covalent van der Waals forces without chemical grafting or covalent tethering. Consequently, the pristine nanotube surfaces fail to conformationally immobilize or pin the polymer chains; instead, the loose interfacial packing and localized disruption of regular polymer chain entanglement near the nanotube boundaries facilitate localized chain slippage and segmental dynamics. Together, the plasticizing effect of PEG 400 and the expanded free volume induced by non-bonded CNT interfaces promote chain mobility at lower thermal energy, leading to the observed decrease in Tg.

Consequently, the concurrent occurrence of enhanced high-temperature pyrolytic endurance (TGA barrier effect) and reduced glass transition temperature (PEG-assisted plasticization and matrix free-volume expansion) is physically coherent and aligns with reported observations for surfactant-assisted CNT/PLA nanocomposite systems.

### 3.4. Microstructure

**Figure 8:**
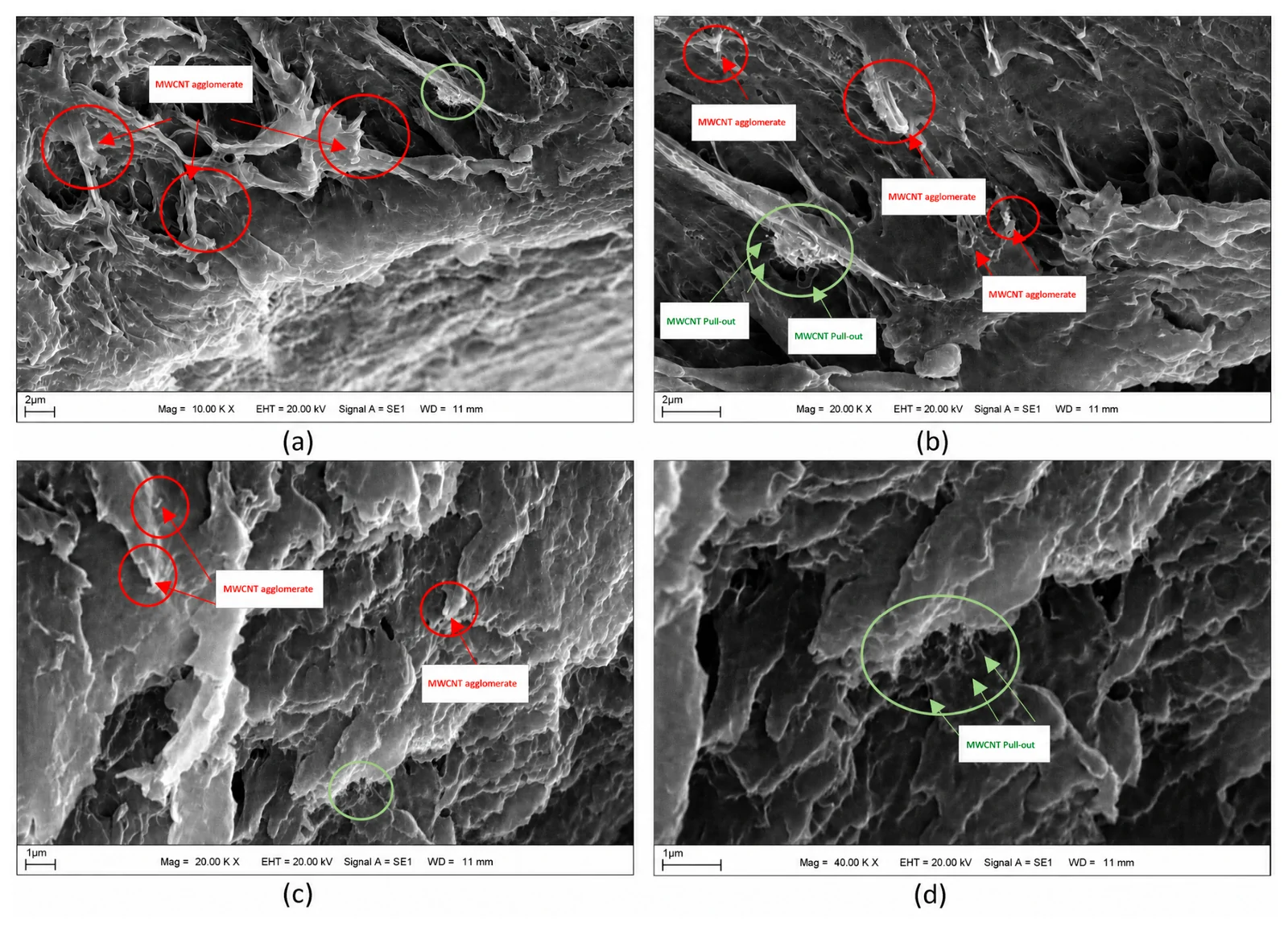
SEM micrographs obtained from the fracture surfaces of the Au-Pd-coated 2 wt.% MWCNT/PLA composite: (**a**,**c**) General fracture surface topography showing localized microscale contrast features. (**b**,**d**) Higher-magnification views of matrix micro-fibrillar deformation and localized filler-rich regions. (Red circles and arrows indicate MWCNT agglomerates, while green circles and arrows indicate MWCNT pull-out regions).

The SEM images of the fracture surfaces of the MWCNT-reinforced PLA composite reveal an irregular and rough morphology with multi-stage fracture features, micro-voids, and localized matrix deformation. As shown in Figure 8, the observed surface topography suggests that the fracture process was influenced by the presence of the MWCNT-containing phase. However, the SEM images do not allow the direct identification of individual MWCNTs or individual nanotube pull-out events within the PLA matrix. Therefore, the elongated features observed in some regions are interpreted conservatively as localized polymer-matrix fibrillation and deformation features formed during fracture, rather than as directly observed nanotubes or nanotube pull-out structures. This interpretation is consistent with the difficulty of directly resolving nanoscale filler–matrix deformation mechanisms from conventional SEM images of polymer nanocomposites [36].

To provide a quantitative estimate of the visible clustering, digital image thresholding and particle-area analysis were performed using ImageJ (Version 1.54g; National Institutes of Health, Bethesda, MD, USA) on representative SEM images. The discernible clustered regions occupied an estimated area fraction of 2.8 ± 0.6%, and their equivalent dimensions predominantly ranged from 0.4 to 1.8 μm. These values refer only to microscale contrast features that could be distinguished in the SEM images and should not be interpreted as a direct measurement of individual MWCNTs. The analysis indicates that pronounced microscale clustering was localized rather than dominant throughout the examined regions. The tendency of MWCNTs to form localized clusters is generally associated with their high aspect ratio, large specific surface area, and strong van der Waals interactions [37]. The observed microscale heterogeneity may influence the mechanical and electrical responses of the composite. Localized filler-rich regions can contribute to variations in stress transfer and conductive-path formation, whereas the surrounding polymer matrix governs the visible fracture morphology. Accordingly, the SEM observations support the presence of localized microscale heterogeneity in the composite, but they do not provide direct evidence of individual MWCNTs, individual nanotube pull-out, or the complete nanoscale dispersion state.

### 3.5. Electrical Conductivity

**Figure 9:**
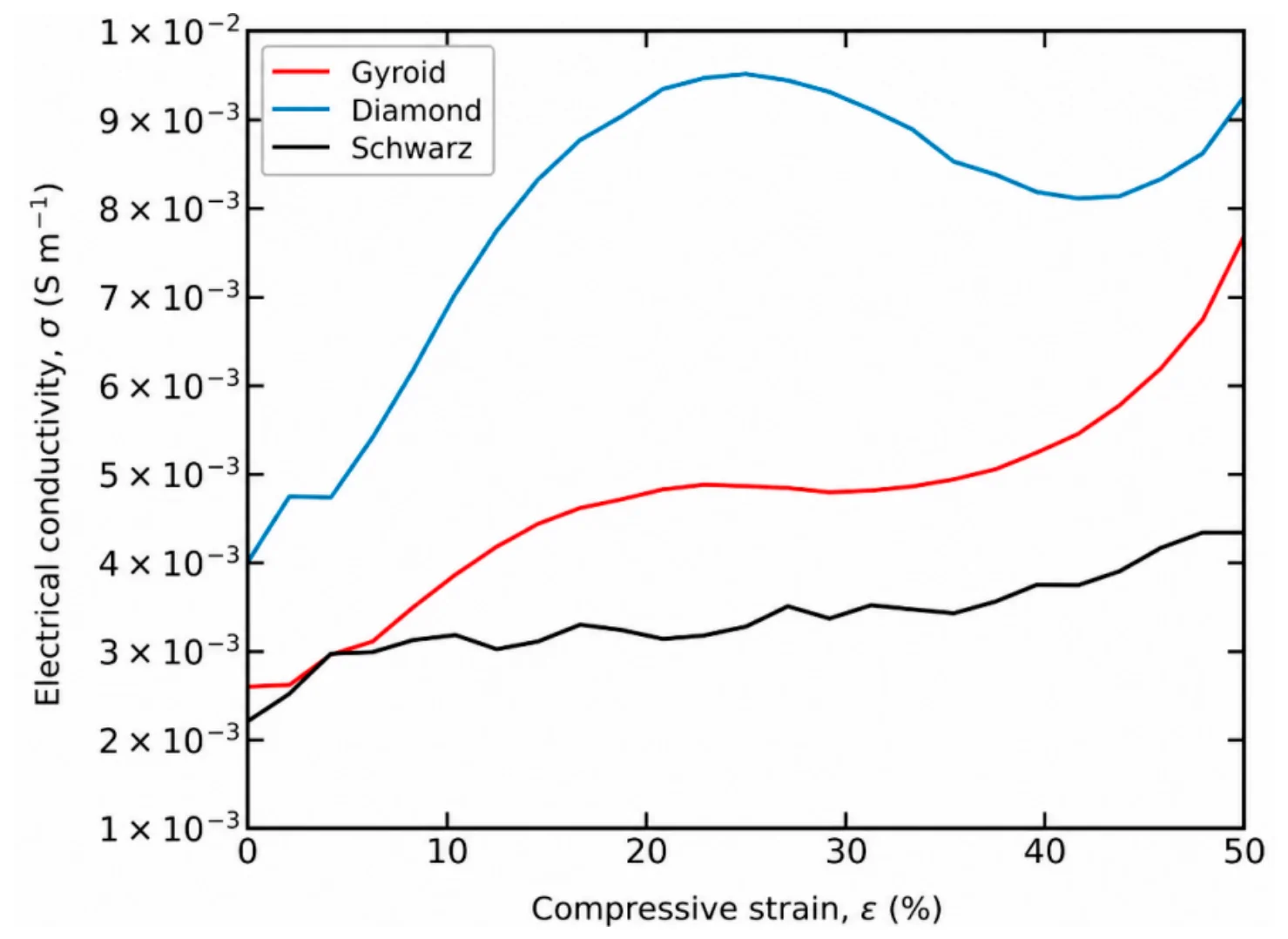
Electrical Conductivity–Strain Curves of TPMS Lattice Structures.

The changes in electrical conductivity of Gyroid, Diamond, and Schwarz Primitive TPMS lattice structures as a function of compressive strain are given in Figure 9. The initial baseline electrical conductivity (reported as mean ± standard deviation, *n* = 3) was (4.02 ± 0.18) × 10^−3^ S m^−1^ for the Diamond structure, (2.61 ± 0.14) × 10^−3^ S m^−1^ for the Gyroid structure, and (2.23 ± 0.11) × 10^−3^ S m^−1^ for the Schwarz Primitive structure, respectively. One-way ANOVA demonstrated that the variation in initial electrical conductivity across the three architectures was statistically significant (F = 124.88, *p* < 0.001), and post-hoc comparisons confirmed significant differences between all topological pairings (*p* < 0.01). Therefore, topological geometry had a statistically validated, decisive effect on the initial macroscopic electron-transport pathways. In the Diamond structure, conductivity increased up to approximately 25% strain and reached a maximum of 9.5 × 10^−3^ S m^−1^. At subsequent strain levels, conductivity decreased to approximately 8.1 × 10^−3^ S m^−1^ and increased again to 9.3 × 10^−3^ S m^−1^ at 50% strain. This fluctuating behavior indicates that the formation of conductive contacts and local cell collapse or changes in the contact configuration occurred simultaneously during compression. In the Gyroid structure, conductivity generally exhibited an increasing trend. Within the approximately 0–25% strain range, conductivity increased from 2.6 × 10^−3^ S m^−1^ to 4.9 × 10^−3^ S m^−1^; a limited plateau/fluctuation region was observed between 25% and 30% strain. As a result of the increase accelerating again at high strain levels, conductivity reached approximately 7.7 × 10^−3^ S m^−1^ at 50% strain. This continuously increasing response indicates that the Gyroid geometry gradually forms new conductive contacts with deformation and has the potential for a more monotonic piezoresistive response. The Schwarz Primitive structure exhibited the lowest conductivity values throughout the entire strain range. Conductivity increased from approximately 2.2 × 10^−3^ S m^−1^ at 0% strain to approximately 4.3 × 10^−3^ S m^−1^ at 50% strain. However, the small fluctuations in the curve suggest that the local and progressive cell collapse observed in the Schwarz Primitive topology affects the continuity of the conductive network.

While the Diamond topology offers the highest initial and maximum conductivity values, the Gyroid structure exhibits a more regular increase in conductivity that becomes more pronounced in the high-strain region. Although the Schwarz Primitive topology possesses lower electrical conductivity, it shows an electrical response sensitive to compressive deformation. These results reveal that the piezoresistive performance of MWCNT/PLA-based TPMS structures is controlled not only by the conductive properties of the composite but also by the effect of the cell topology on the conductive network under deformation.

The electrical percolation threshold is the minimum MWCNT content required to form a continuous conductive network within the PLA matrix. The threshold value depends on several factors, including nanotube distribution, processing method, and nanotube aspect ratio. In this study, the composite contained 2 wt.% MWCNT and all TPMS structures exhibited measurable initial electrical conductivity. The initial conductivity values were (4.02 ± 0.18) ×10^−3^ S m^−1^ for Diamond, (2.61 ± 0.14) ×10^−3^ S m^−1^ for Gyroid, and (2.23 ± 0.11) ×10^−3^ S m^−1^ for Schwarz Primitive. These results confirm that the MWCNT content was above the effective electrical percolation threshold of the composite and that a continuous conductive network was formed. This conductive network enabled the resistance changes to be measured during compression and allowed the TPMS structures to exhibit piezoresistive behavior.

### 3.6. Piezoresistive Behaviors of TPMS Lattice Structures

**Figure 10:**
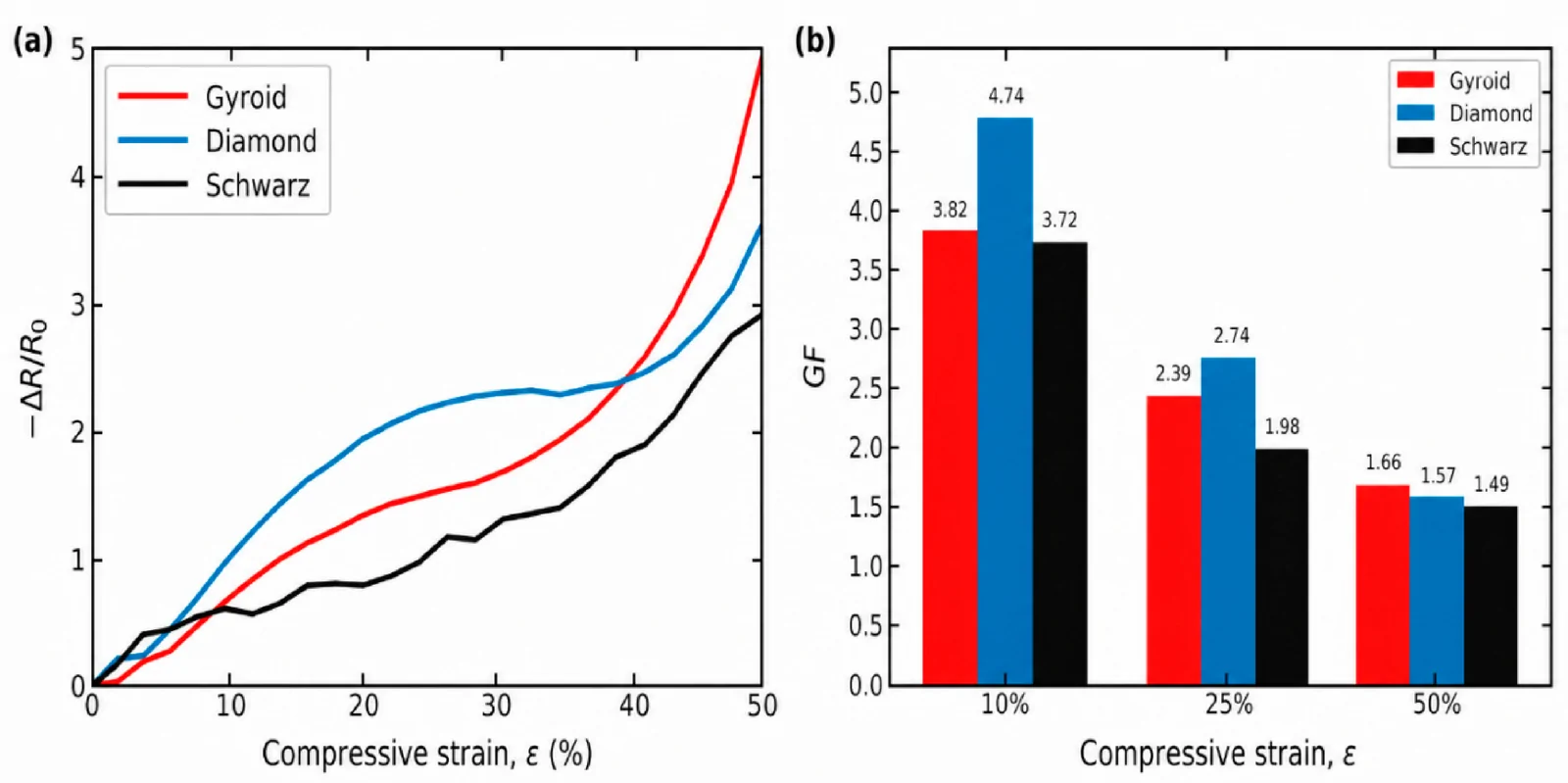
(**a**) Relative resistance change-compressive strain curves of TPMS lattice structures. (**b**) Gauge factor.

The relative resistance changes of TPMS lattice structures with Gyroid, Diamond, and Schwarz Primitive topologies as a function of compressive strain are given in Figure 10a. In all topologies, electrical resistance decreased with increasing compressive strain; accordingly, the −*ΔR/R*_0_ value increased. This behavior is associated with the conductive phases containing MWCNT approaching each other during compressive deformation, the increase in contact points between nanotubes, and the formation of more effective conductive paths for electron transport. Therefore, it is observed that the produced MWCNT/PLA-based TPMS structures exhibit a distinct piezoresistive response under compressive loading. The Diamond topology exhibited the highest relative resistance change in the low- and medium-strain regions. At approximately 10% strain, the structure showed a faster decrease in resistance compared to the other topologies, and this trend continued up to 25% strain. This behavior of the Diamond structure can be explained by the deformation having a more pronounced effect on the conductive network due to its high initial stiffness and effective load transfer mechanism. However, the limited increase rate of the Diamond curve at higher strain levels is associated with partial densification of the cells and the conductive contact network approaching saturation at a certain level. Although the Gyroid topology exhibited a lower relative resistance change than the Diamond structure in the low-strain region, it showed a more pronounced increase after approximately 35–40% strain. At 50% strain, the −*ΔR/R*_0_ value of the Gyroid structure reached approximately 5, showing the highest relative resistance change among the investigated topologies. This result indicates that the continuous and curvilinear cell architecture of the Gyroid structure enables the gradual formation of new conductive contacts at high deformation levels. In addition, the regular increasing trend of the Gyroid topology at high strains indicates that it may provide an advantage for pressure or deformation sensor applications with a wide operating range. The Schwarz Primitive topology exhibited a lower relative resistance change throughout the entire strain range. The local fluctuations observed in the curve are associated with temporary changes in the continuity of the conductive network as a result of local buckling and progressive collapse of the cells during the compression test. Nevertheless, the general increase in resistance changes with strain in the Schwarz Primitive structure confirms that this topology also has piezoresistive sensing capability.

The compression gauge factor (GF) values given in Figure 10b reveal that the strain sensitivities of the topologies differ depending on the strain level. At 10% strain, the Diamond structure exhibited the highest sensitivity with a GF of approximately 4.74; the GF values of the Gyroid and Schwarz Primitive structures were determined as approximately 3.82 and 3.72, respectively. At 25% strain, the Diamond topology maintained the highest sensitivity with a GF value of approximately 2.74, while values of approximately 2.39 and 1.98 were obtained for the Gyroid and Schwarz Primitive structures, respectively. In contrast, at 50% strain, the Gyroid topology exhibited higher sensitivity with a GF of approximately 1.66 than the Diamond (1.57) and Schwarz Primitive (1.49) structures. It is pertinent to delineate the electromechanical regime investigated herein: the piezoresistive characterization was conducted under monotonic uniaxial compression up to 50% strain to elucidate the baseline deformation mechanics, conductive network densification, and intrinsic topological sensitivity limits spanning both the pre-yield and post-yield consolidation regimes. Although the observed electromechanical attenuation was continuous, monotonic, and devoid of transient open-circuit instabilities across all topologies, attributing operational “cyclical stability” under large strains would be physically untenable without cyclic hysteresis data. Owing to the semi-rigid viscoelastic-plastic nature of the PLA matrix, compressive deformation beyond the linear elastic threshold (approximately *ε* > 0.10) inevitably induces plastic hinge formation, localized cell-wall buckling, and irreversible structural collapse, which inherently preclude full geometric and electromechanical recovery upon complete unloading. Consequently, the monotonic large-strain sensing capability (*ε* = 0.10–0.50) demonstrated by these architected lattices is predominantly tailored for single-event impact detection, structural integrity threshold monitoring, and irreversible energy-absorbing sensor components. Conversely, reversible and repeatable continuous sensing requires operation strictly confined within the low-strain elastic regime (*ε* ≤ 0.05–0.10), wherein microstructural recovery remains preserved. Quantitative cyclic loading–unloading durability, dynamic hysteresis, and gauge recovery across iterative cycles constitute a vital scope for subsequent investigations.

## 4. Conclusions

This study demonstrates that, under identical material composition, relative density, and processing conditions, TPMS unit-cell topology governs the mechanical, electrical, and piezoresistive performance of MWCNT/PLA lattice structures. The major inferences drawn from this work are summarized below:

Topology as an independent design factor: Unit-cell geometry, rather than material composition alone, is a statistically significant and independent determinant of both mechanical and electrical response, confirming that lattice architecture can be used as a design lever for multifunctional performance tuning.

Diamond topology for load-bearing sensing: The dual-strut, multi-directional load path of the Diamond topology provides superior stiffness and energy absorption together with high piezoresistive sensitivity at low-to-medium strains, making it suitable for load-bearing structures requiring early-stage damage or strain detection.

Gyroid topology for wide-range sensing: The continuous, zero-mean-curvature surface of the Gyroid topology promotes a more gradual and monotonic evolution of the conductive network, resulting in superior sensitivity at high strains and making it preferable for applications requiring strain monitoring over a broad deformation range.

Schwarz Primitive as the least favorable topology: The flat-wall geometry of the Schwarz Primitive topology leads to localized stress concentrations and early cell collapse, consistently limiting its mechanical and sensing performance relative to the Diamond and Gyroid topologies.

Application potential and future work: These findings position MWCNT/PLA TPMS lattices as promising candidates for lightweight smart structures, impact-energy-absorbing components, and structural health-monitoring systems. As the present study was limited to monotonic compression, future work should address cyclic loading durability, hysteresis behavior, and long-term repeatability to establish the operational reliability of these architectures under real-world sensing conditions.

## Figures and Tables

**Table 1:** Materials used for composite filament fabrication.

Material	Role	Specifications
Polylactic Acid	Polymer matrix	Density: 1.24 g/cm^3^; melting temperature (Tm): 150–180 °C; glass transition temperature (Tg): 55–65 °C
Multi-Walled Carbon Nanotubes	Conductive filler	Purity > 96%; outside diameter: 8–18 nm
Dichloromethane	Solvent	Purity ≥ 99.9%; molecular weight: 84.93 g/mol; density: 1.33 g/cm^3^ (20 °C)
Polyethylene Glycol 400	Dispersing agent	PEG 400

**Table 2:** Design parameters of TPMS lattice structures.

Architecture	Trigonometric Function	Relative Density (RD)	Total Volume (Vt) (mm^3^)	Solid Volume (Vs) (mm^3^)	Unit Cell Size (mm)	Thickness (mm)	3-D Model
**Gyroid**	$G:\sin\left(x\right).\cos\left(y\right)$ $+\sin\left(y\right).\cos\left(z\right)$ $+\sin\left(z\right).\cos\left(x\right)=0$	30%	6144	1843	4	0.60	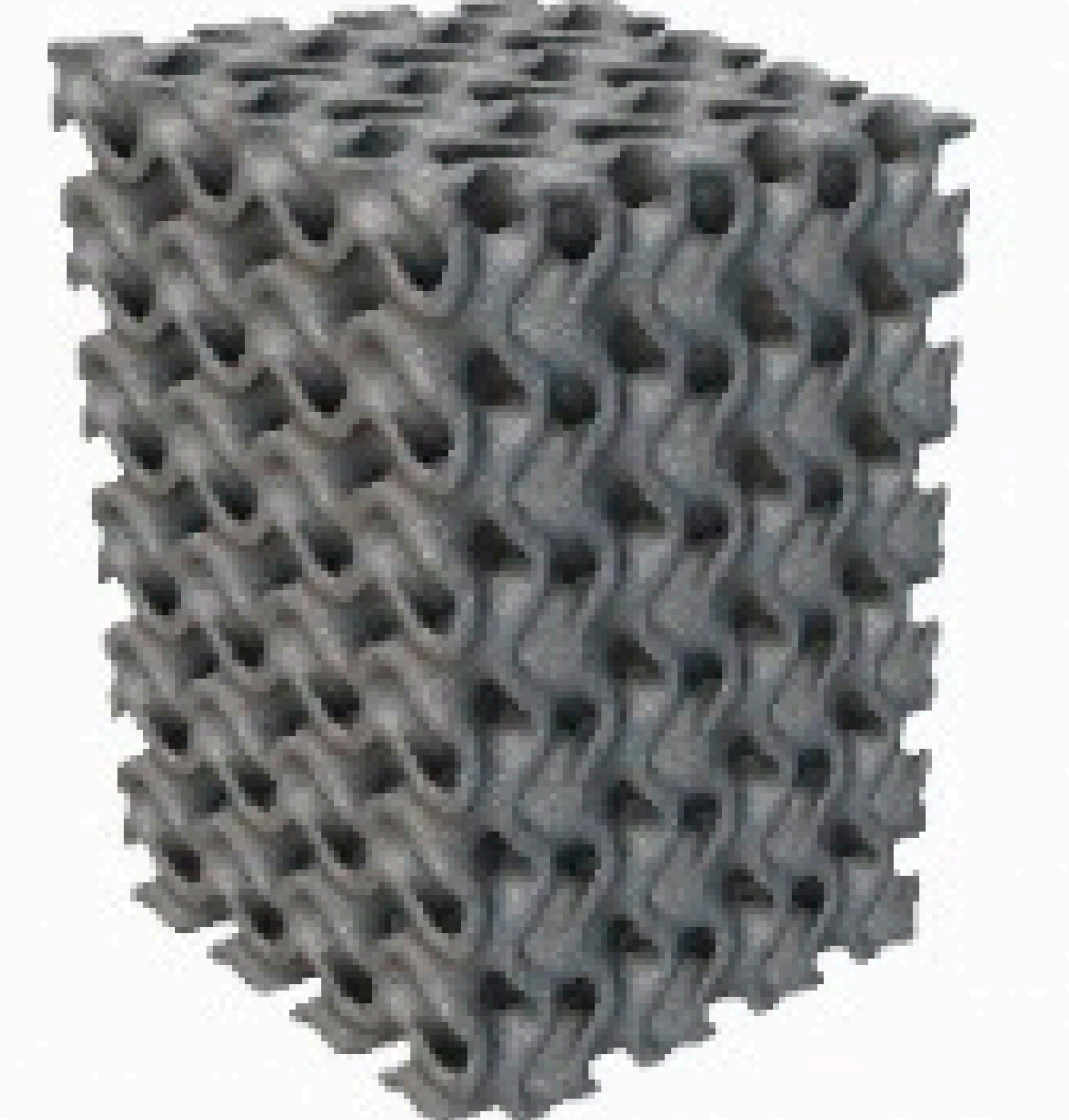
**Diamond**	$D:\sin\left(x\right).\sin\left(y\right).\sin\left(z\right)\;+\sin\left(x\right).\cos\left(y\right).\cos\left(z\right)\;+\cos\left(x\right).\sin\left(y\right).\cos\left(z\right)\;+\cos\left(x\right).\cos\left(y\right).\sin\left(z\right)=0$					0.48	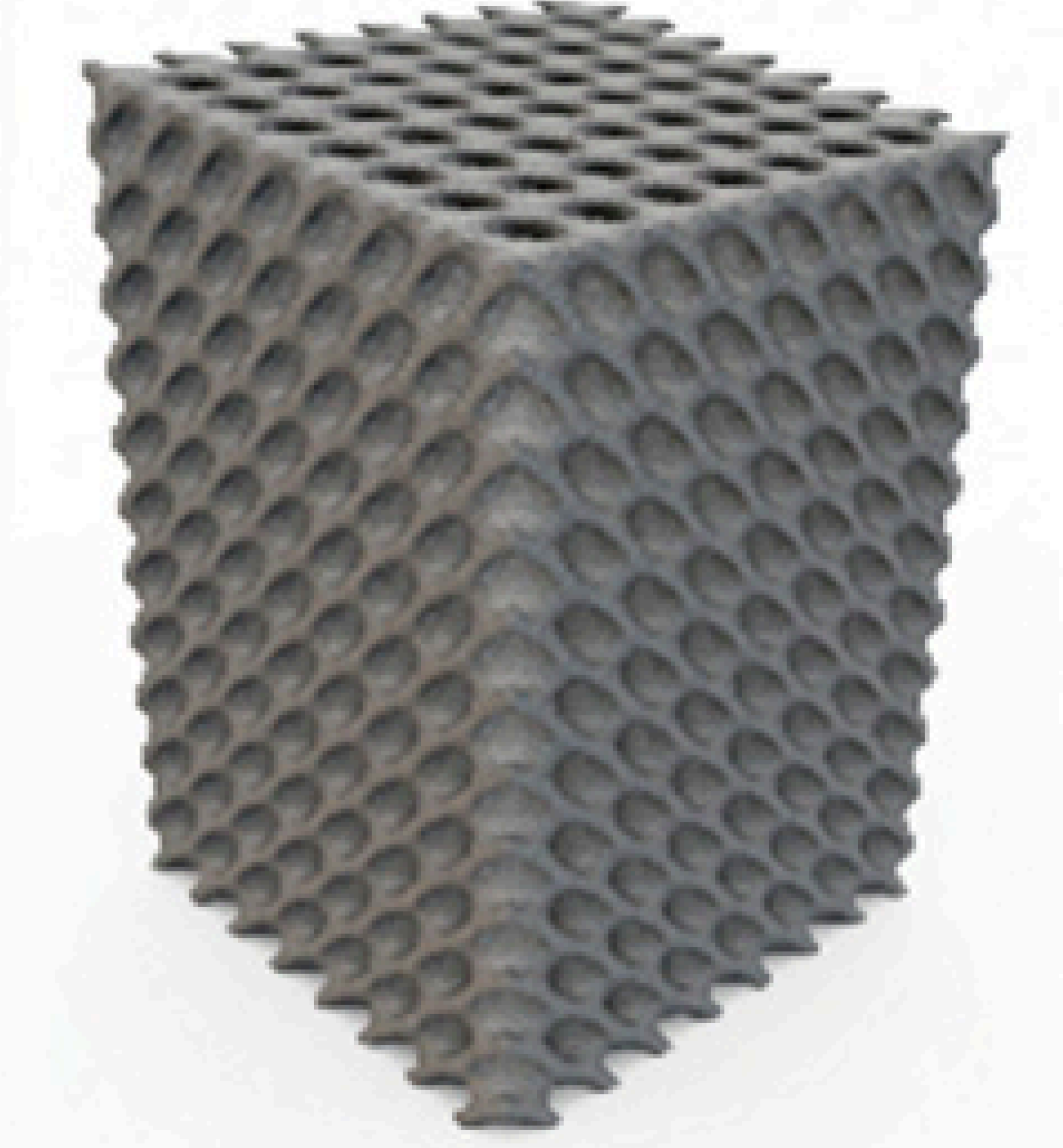
**Schwarz Primitive**	$P:\cos\left(x\right)+\cos\left(y\right)+\cos\left(z\right)=0$					0.68	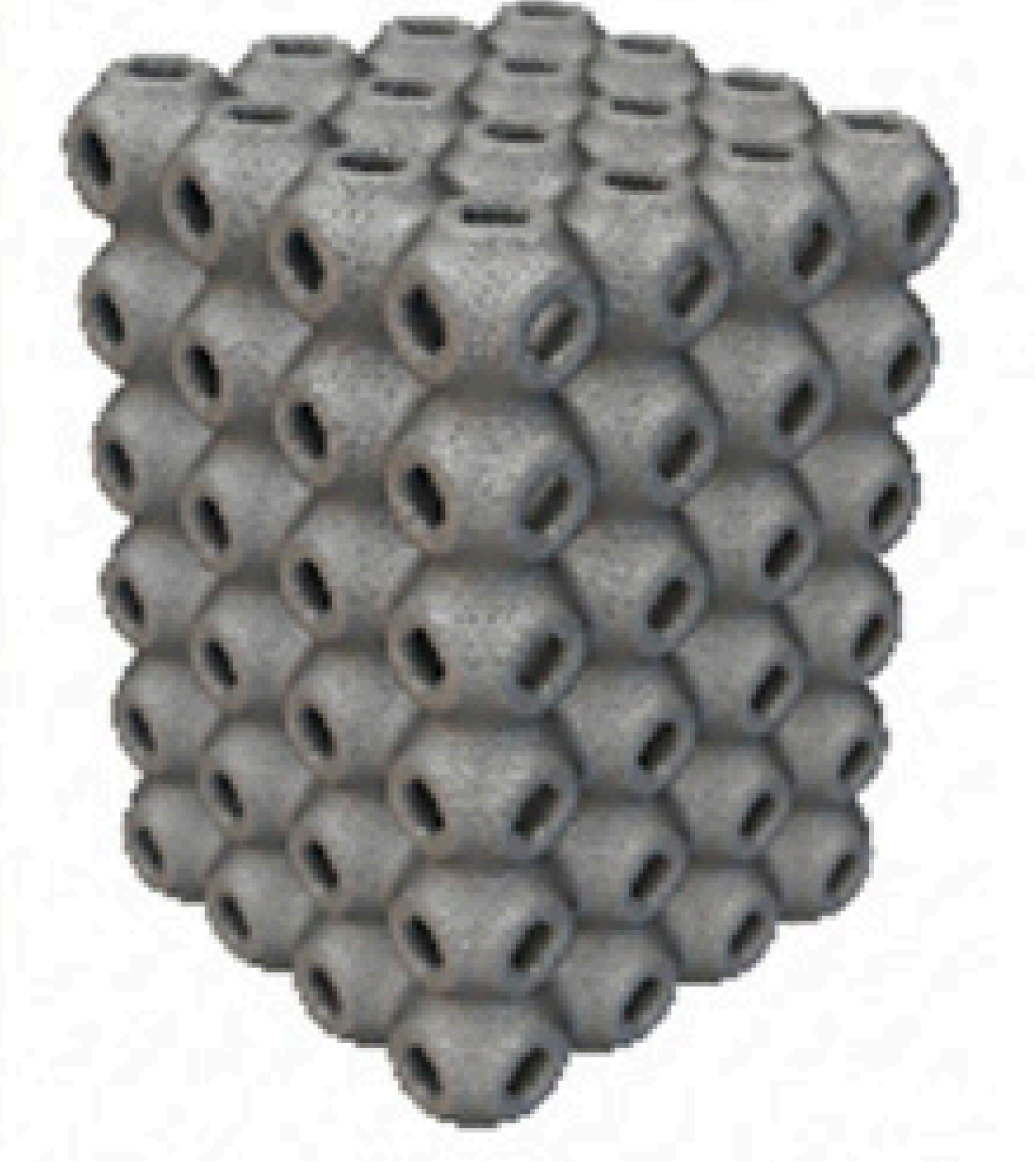

**Table 3:** FFF printing parameters.

Parameter	PLA + MWCNT
Extruder temperature, °C	220
Bed temperature, °C	60
Nozzle diameter, mm	0.4
Printing speed, mm/s	60
Layer height, mm	0.2

**Table 4:** Mass, compressive stress, specific energy absorption (SEA), and Shore D hardness values of TPMS lattice structures with Gyroid, Diamond, and Schwarz Primitive topologies (mean ± standard deviation, *n* = 3).

TPMS Structure	Mass (g)	Compressive Stress (MPa)	SEA at 50% Strain (J g^−1^)	Shore D Hardness
**Gyroid**	2.95 ± 0.025	8.777 ± 0.327	6.149 ± 0.199	73.5 ± 0.5
**Diamond**	3.40 ± 0.200	14.164 ± 0.164	9.161 ± 0.191	75.6 ± 0.2
**Schwarz** **Primitive**	2.48 ± 0.080	3.152 ± 0.202	2.917 ± 0.147	71.2 ± 2.2

**Table 5:** Experimental FTIR absorption bands and vibrational mode assignments for neat PLA, MWCNTs, and 2 wt.% MWCNT/PLA nanocomposite.

Material	Wavenumber (cm^−1^)	Vibrational Mode Assignment	Chemical Structure/Group
**Pure PLA**	2995, 2946	Asymmetric & symmetric stretching	Aliphatic –CH–, –CH_3_
	1748	Strong stretching vibration	Ester carbonyl (C=O)
	1452	Asymmetric bending (deformation)	–CH_3_ methyl
	1382, 1360	Symmetric bending/ deformation	–CH–, –CH_3_
	1182, 1128	Asymmetric stretching vibration	Ester C–O–C
	1084	Symmetric stretching vibration	Ester C–O–C/C–O
	1044	Stretching vibration	C–CH_3_
	868	Skeletal C–C stretching	Polymer backbone
	754	C–C bending/crystalline phase	Amorphous/crystalline ring
**MWCNTs**	2918	Weak stretching vibration	Trace C–H defects
	1530	Skeletal vibration	Graphitic aromatic C=C
**2 wt.% MWCNT/PLA**	2996, 2947	Main chain stretching (unaltered)	Aliphatic C–H
	1748	Carbonyl stretching (slightly broadened)	Ester C=O
	1452, 1360	Bending vibrations (unaltered)	–CH_3_ deformation
	1182, 1084	Ester bond stretching (retained)	Ester C–O–C
	868, 754	Skeletal vibrations (retained)	Main chain backbone

## Data Availability

The original contributions presented in this study are included in the article. Further inquiries can be directed to the corresponding author.
